# Trans‐Omics Integration Reveals That the Kidney Contributes to Systemic Aging via Sexually Dimorphic Accumulation of Glycosphingolipids

**DOI:** 10.1002/mco2.70669

**Published:** 2026-03-07

**Authors:** Zhen Ni, Chenyin Cao, Yanlin Tian, Jinming Mu, He Tian, Zehua Wang, Shaohua Zhang, Mingjun Cao, Yuntian Yang, Wei Ling Florence Lim, Jingkang Cui, Huan Sun, Huan Miao, Yuan Wang, Jie Du, Timothy Kwok, Huan Chen, Sin Man Lam, Guanghou Shui

**Affiliations:** ^1^ State Key Laboratory of Molecular Developmental Biology, Institute of Genetics and Developmental Biology, Chinese Academy of Sciences Beijing China; ^2^ Beijing Life Science Academy Beijing China; ^3^ University of Chinese Academy of Sciences Beijing China; ^4^ Guangzhou National Laboratory Guangzhou China; ^5^ Jiangsu Provincial Key Laboratory of Molecular Targets and Intervention for Metabolic Diseases LipidALL Technologies Company Limited Changzhou China; ^6^ Beijing Anzhen Hospital Capital Medical University Beijing China; ^7^ Department of Medicine and Therapeutics The Chinese University of Hong Kong, Prince of Wales Hospital Shatin China; ^8^ Guangzhou Institute of Cancer Research, the Affiliated Cancer Hospital Guangzhou Medical University Guangzhou China

## Abstract

Age‐associated deterioration of physiological functions occurs at heterogeneous rates across individual organs. A granular evaluation of systemic metabolic mediators of aging in a healthy human cohort (*n* = 225) identified prominent increases in circulating uremic toxins, a finding recapitulated in mice. We connected these systemic aging profiles to renal metabolism, specifically linking glucosylceramide (GluCer) accretion to renal functional decline at late middle‐age that coincides with the temporal surge in uremic toxins. Importantly, age‐associated increases in circulating GluCer, largely contributed by the kidneys, are conserved from mice to humans, and are significantly associated with enhanced risk of multiple causes of mortality in aged individuals. We further showed that GluCer accumulation, commencing in late middle‐age of females, impairs mitophagy via disrupting mitochondria–lysosome untethering, and undermines mitochondrial respiratory function via purine‐dependent activation of mTORC1 signaling that can be rescued by pharmacological purine depletion. The resulting age‐associated renal dysfunction is female‐biased, likely due to sexually dimorphic GluCer handling. Our work provides a molecular basis for the sex‐specific benefits of mTOR inhibition on lifespan, and highlights clinically relevant inhibitors of purine metabolism as potential senotherapeutics to mitigate kidney‐driven systemic aging.

## Graphical abstract

**Female‐specific renal GluCer accumulation disrupts mitochondrial quality control via a conserved purine‐mTORC1 pathway, triggering a wave of uremic toxins into the systemic circulation that constitutes a female‐biased vulnerability toward renal‐driven multiorgan senescence**. As estrogen levels fall in late middle‐age, GluCer accumulates in female kidneys. Elevated purines activate mTOR, disrupt mitophagy, suppress PGC1α, and impair mitochondrial–lysosome untethering, leading to mitochondrial dysfunction and reduced ATP production. Renal failure promotes systemic accumulation of uremic toxins and multiorgan senescence. Purine inhibition attenuates mTOR activation and restores mitochondrial homeostasis, highlighting a potential senotherapeutic strategy.

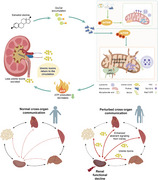

## 1 Introduction

Medical advancements have significantly improved the quality of life and substantially extended the average human lifespan. Concurrently, aging‐related diseases have emerged as major public health concerns. Aging represents the greatest common risk factor for chronic diseases [1], while age‐related functional decline of different organs denotes commonalities underlying various chronic diseases. The trajectory of age‐related deterioration in function varies for individual organs, and biological aging clocks specific to distinct organ systems have been established [2]. A multiorgan characterization of aging phenomes coupled with the identification of systemic metabolic mediators of aging is, therefore, expected to unravel interorgan crosstalk that coordinates the overall aging process of the organism as an entity.

Lipids, as integral structural components of mammalian systems, partake in various biochemical signaling processes that underpin cellular and tissue function [3]. Preceding studies have implicated lipids in the pathogenesis of various age‐related diseases, such as cardiovascular disease [4, 5], diabetes [6, 7], Alzheimer's disease [8, 9, 10], and chronic kidney disease (CKD) [11, 12]. As examples, lower levels of lysophosphatidylcholines (LPCs) were associated with renal failure in CKD [11], while changes in phosphatidylcholines and triacylglycerols were correlated with higher CKD risk [12].

On top of lipids, polar metabolites constitute an added dimension of the biological phenome that together confer the closest readout of the cellular phenotype. Metabolomic profiling of aging cohorts has identified several metabolites from, for example, the categories of carbohydrates, amino acids, and nucleotides, which were associated with the aging phenotype [13, 14]. Omics‐driven approaches are useful in offering a granular view of the metabolic landscape that facilitates the discovery of novel functional metabolites under different biological contexts [15]. Analytical challenges, such as limited metabolite identification and imprecise quantification, however, can substantially impede the broader application of metabolomics in aging research and undermine the validity of age‐related metabolite markers [16].

In this study, we first utilized precise, quantitative metabolomic approaches [16] to establish an array of systemic, age‐related metabolite alterations in the human plasma of a cross‐sectional cohort. These systemic metabolic mediators were recapitulated during the aging process in mice, which enabled us to leverage the murine model to uncover the contributory roles of different organ systems toward the systemic metabolic perturbations observed in human aging. Using high‐coverage, quantitative lipidomic profiling [17] of metabolic changes in five major peripheral organs and/or tissues in mice, our findings untangle metabolic signaling pathways crucial to preserving kidney function, which contribute positively to human health, and unveil new intervention targets to promote healthy aging.

## 2 Results

### 2.1 Overlapping Metabolic Features Between Human Systemic Aging and Compromised Renal Function

Using a robust, nontargeted strategy for accurate quantitation and precise profiling of 480 metabolites developed in‐house [16], we investigated the aging‐associated plasma metabolomes in a cross‐sectional cohort of 225 ostensibly healthy individuals aged between 20 and 88 years (Table 1). Linear regression analysis uncovered an array of 280 plasma metabolites associated with aging. Over 70% (200/280) of these age‐associated metabolites were positively correlated with increasing age, which predominantly consisted of fatty acyls, carboxylic acids/derivatives, and glycerophospholipids (Table S1; Figure S1A). Age‐associated accretions in plasma acylcarnitines, which were inversely associated with estimated glomerular filtration rate (eGFR) [18], indicate declining renal function with aging. The kidney plays a key role in carnitine biosynthesis from lysine and methionine, and in carnitine excretion into the urine and plasma [19]. Pathway enrichment analysis of age‐related metabolites uncovered several pathways related to urea metabolism, including “urea cycle and metabolism of amino groups”, “biomarkers for urea disorders”, and “urea cycle and associated pathways” (Figure S1B). The involvement of urea metabolism suggests perturbations in hepatic and/or renal function with aging [20]. A gross comparison between the age‐associated plasma metabolite changes reported here with preceding metabolome perturbations in CKD revealed significant overlap, such as aberrant levels of short and medium‐chain acylcarnitines [12, 21, 22, 23], gut microbiota metabolites [23], tryptophan metabolites [24], purine metabolites [25, 26], acetylated amino acids [27, 28, 29], bile acids [23], LPCs [23, 30], and lysophosphatidylethanolamines (LPEs) [23, 31] (Table S1).

**TABLE 1:** Clinical characteristics of normative aging cohort.

Characteristic	Statistics, *N* = 225
Sex (female), *n* (%)	114 (50.7%)
Age (years)	
Mean (SD)	52.56 (18.00)
Min–max	20.00–88.00
Median (Q1, Q3)	52.00 (38.00, 68.00)
BMI (kg/m^2^)	
Mean (SD)	23.73(3.64)
Min–max	14.61–36.41
Median (Q1, Q3)	23.89 (21.35, 25.77)
Unknown	57 (25.3%)
History of hypertension, yes (%)	39 (17.3%)
Unknown	55 (24.4%)
History of coronary heart disease, yes (%)	13 (5.8%)
Unknown	55 (24.4%)
History of diabetes, yes (%)	12 (5.3%)
Unknown	55 (24.4%)
History of dyslipidemia, yes (%)	39 (17.3%)
Unknown	55 (24.4%)
History of myocardial infarction, yes (%)	0 (0%)
Unknown	55 (24.4%)
History of drinking alcohol, *n* (%)	27 (12%)
Unknown	55 (24.4%)
SBP (mmHg)	
Mean (SD)	123.03 (16.70)
Min–max	82.00–173.00
Median (Q1, Q3)	120.00 (111.00, 133.50)
Unknown	58 (25.8%)
DBP (mmHg)	
Mean (SD)	72.87 (11.61)
Min‐Max	3.00–102.00
Median (Q1, Q3)	72.00 (65.00,78.75)
Unknown	59 (26.2%)
TG (mmol/L)	
Mean (SD)	1.25 (0.83)
Min–max	0.24–6.48
Median (Q1, Q3)	1.07 (0.79,1.45)
Unknown	57 (25.3%)
TC (mmol/L)	
Mean (SD)	4.73 (0.84)
Min–max	2.88–7.11
Median (Q1, Q3)	4.65 (4.10,5.26)
Unknown	57 (25.3%)
HDL (mmol/L)	
Mean (SD)	1.41 (0.35)
Min–max	0.78–2.70
Median (Q1, Q3)	1.38 (1.17, 1.61)
Unknown	57 (25.3%)
LDL (mmol/L)	
Mean (SD)	2.81 (0.73)
Min–max	1.27–5.08
Median (Q1, Q3)	2.70 (2.27, 3.29)
Unknown	57 (25.3%)
CREA (µmol/L)	
Mean (SD)	64.85 (14.72)
Min–max	35.30–116.00
Median (Q1, Q3)	64.15 (54.50, 72.35)
Unknown	53 (23.6%)
BUN (mmol/L)	
Mean (SD)	4.93 (1.36)
Min–max	2.40–9.50
Median (Q1, Q3)	4.70 (3.90, 5.83)
Unknown	53 (23.6%)
UA (µmol/L)	
Mean (SD)	323.23 (76.17)
Min–max	142.20–573.90
Median (Q1, Q3)	321.00 (269.53, 378.23)
Unknown	53 (23.6%)
RI (mU/L)	
Mean (SD)	7.68 (6.84)
Min–max	1.90–81.50
Median (Q1, Q3)	6.80 (5.20, 8.60)
Unknown	80 (35.6%)
GLU (mmol/L)	
Mean (SD)	5.32(0.73)
Min–max	3.92–8.60
Median (Q1, Q3)	5.16 (4.88, 5.57)
Unknown	57 (25.3%)

Abbreviations: BMI, body mass index; BUN, urea nitrogen; CREA, creatinine; DBP, diastolic blood pressure; GLU, glucose; Hcy, homocysteine; HDL, high‐density lipoprotein; LDL, low‐density lipoprotein; HSCRP, hypersensitive‐c‐reactive‐protein; RI, regular insulin; SBP, systolic blood pressure; TC, total cholesterol; TG, triglycerides; UA, uric acid.

Standard linear regression analyses might overlook undulating metabolite alterations across aging. To overcome this, we next conducted unsupervised hierarchical clustering to group age‐associated plasma metabolites that possessed similar trajectories of changes. Nine metabolite trajectories were obtained (Table S2; Figure S1C,D), demonstrating the nonlinear nature of several age‐associated metabolite alterations. Classification of metabolite trajectory allows a clear visualization of temporal fluctuations in metabolite levels with age (Figure S1D,E), and metabolites with a common trajectory (i.e., within the same cluster) possibly indicate co‐regulation across aging. We defined the metabolic nature of individual clusters based on the dominant metabolite class. Metabolites relevant to renal function were distributed amongst clusters 2–4, which predominantly comprised lipids, uremic toxins, N‐acetyl‐amino acids (N‐acetyl‐AAs), and acylcarnitines. Clusters 2 and 3 contained metabolites downstream of tryptophan and tyrosine metabolism, such as kynurenic acid, L‐kynurenine, and P‐cresol‐sulfate, which denote uremic toxins normally excreted by the kidneys. Uremic toxins accumulate under impaired kidney function and can inflict damage on multiple organs [26]. Cluster 3 also included several N‐acetyl‐AAs generated from the catabolism of N‐acetylated proteins. Under normal circumstances, N‐acetyl‐AAs are deacetylated and reabsorbed in the kidneys via the amino acid salvage pathway, and their accumulation illustrates compromised renal salvage function [32]. A simple overview of age‐related metabolic alterations thus underscores dysregulated renal function as a key aspect of systemic aging in humans.

### 2.2 DE‐SWAN Analysis Identifies Bursts in Differential Metabolites Across Aging

**FIGURE 1:**
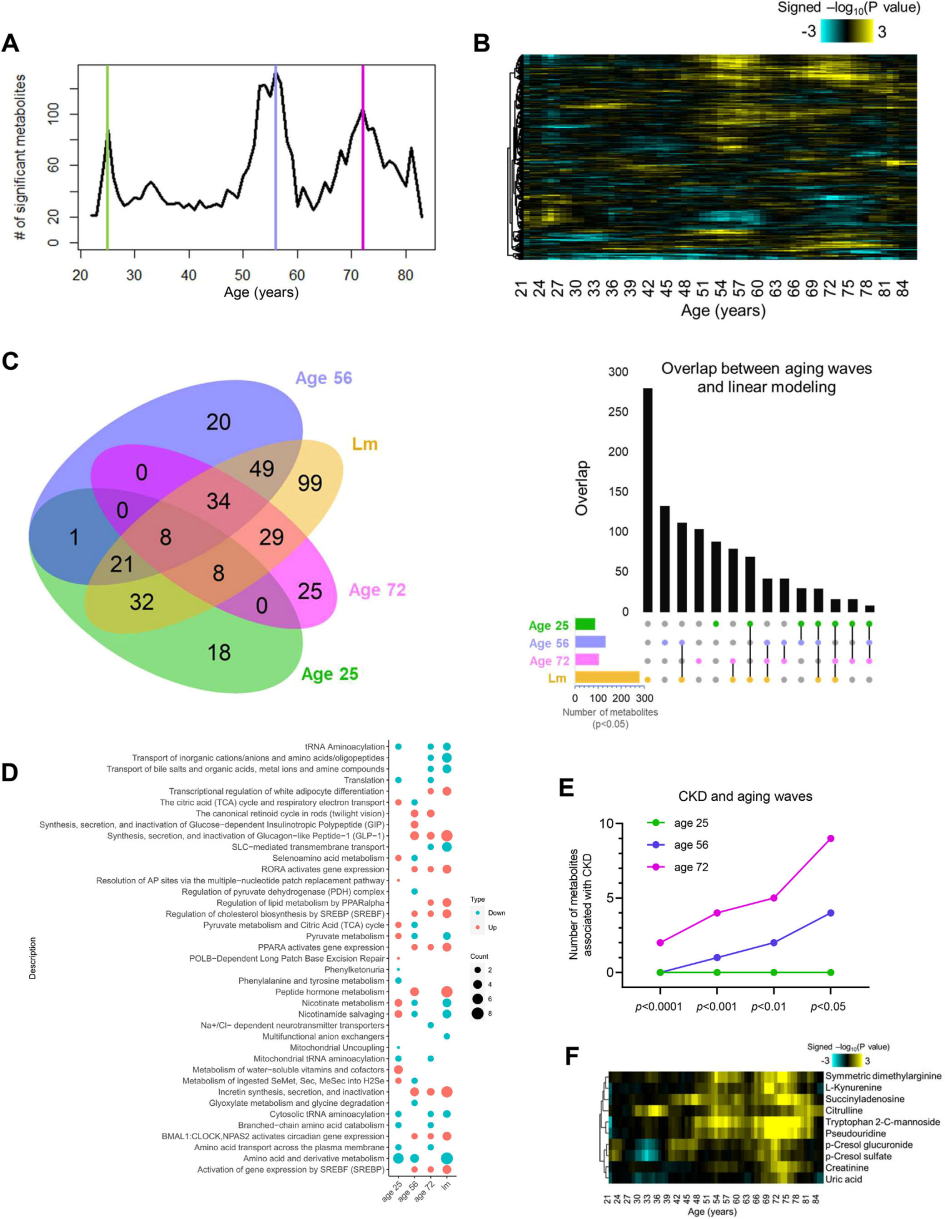
**Metabolic features of human systemic aging coincide with renal functional dysregulation**. (A) Number of plasma metabolites differentially expressed across normative aging (*n* = 225). Three localized peaks in significant age‐associated metabolites at the ages of 25, 56, and 72 years were identified by DE‐SWAN analysis. (B) Waves of aging plasma metabolites characterized by DE‐SWAN analysis. Positive associations are shown in yellow, and negative associations are shown in turquoise. (C) A Venn diagram illustrates intersections between linear modeling (Lm) and age‐associated metabolites at the metabolic crests of 25, 56, and 72 years as uncovered by DE‐SWAN analysis. UpSet plot on the right depicts intersections between sets of metabolites within individual metabolic crests from DE‐SWAN analysis and from linear modeling. (D) Visualization of pathways significantly enriched for aging‐related metabolites identified by linear modeling and DE‐SWAN at age 25, 56, and 72 years (*n* = 225). Dots represent the presence of a significantly enriched pathway for up‐regulated or down‐regulated metabolites. The size of the dot is proportional to the count of metabolites enriched in the pathway. Enrichment was tested using the hypergeometric test (Reactome), *p*‐value cut‐off was set to *p* < 0.05. (E) Line plot displays the numbers of statistically significant CKD‐associated metabolites at individual metabolic crests identified by DE‐DWAN. Aging‐related metabolites at age 25, 56, and 72 years were categorized on the basis of *p*‐values ranging from *p* < 0.0001 to *p* < 0.05. F. Heatmap illustrates the waves of CKD‐associated metabolites characterized by DE‐SWAN. Positive associations are shown in yellow, and negative associations are shown in turquoise.

Since metabolic reactions are intertwined and often buffered by compensatory mechanisms, aging‐associated metabolic phenotypes might be masked until resiliency mechanisms fall apart. Instead of a gradual, linear process, we therefore expect systemic aging to occur in waves from a metabolic perspective. Utilizing differential expression‐sliding window analysis (DE‐SWAN) [33], designed to select quantitative changes in phenotype throughout life, we uncovered three metabolic crests at ages 25, 56, and 72, which corresponded approximately to life stages of young, middle‐aged, and old‐aged in humans (Figure 1A). The DE‐SWAN algorithm analyzes metabolite changes in sliding windows (in increments of 1 year) of 20 years, and compares two groups in parcels of 10 years (e.g., 35–45 years compared with 45–55 years) throughout all ages examined [33]. DE‐SWAN analysis unmasked the sequential effects of aging on the systemic metabolome and revealed several metabolite clusters that were altered in waves across aging (Figure 1B). As anticipated, DE‐SWAN analysis additionally identified 18, 20, and 25 metabolites specifically altered at ages 25, 56, and 72, respectively, which were not revealed by linear regression analysis (Figure 1C; Table S3). To obtain metabolic representation of these age‐related crests, we conducted pathway enrichment analysis based on metabolites within individual clusters (Figure 1D; Table S4). DE‐SWAN analysis detected temporal dysregulation in metabolic pathways otherwise masked in linear modeling. For example, linear regression analysis indicated a general downregulation in “nicotinamide salvaging” and “nicotinamide metabolism” across aging, but DE‐SWAN revealed that these pathways were upregulated at 25 years, then downregulated at 56 years instead (Figure 1D). Enhanced nicotinamide salving in young individuals and its subsequent decline at middle age are in agreement with the reported effect of increasing NAD+ bioavailability on improving healthspan [34]. Importantly, pathways concerning the regulation of organic and/or inorganic ion and amino acid transport mediated by solute carrier proteins (SLC) were downregulated with aging, particularly at 72 years (Figure 1D). Enrichment of these pathways was primarily ascribed to reductions in plasma isoleucine and leucine levels with aging. Indeed, appreciable reduction in plasma isoleucine was previously observed in patients with acute kidney injuries, attributed to abated activity of SLC6a19 neutral amino acid uniporter in proximal renal tubular cells—an early cellular response to kidney injuries resulting from ischemia [35]. Indeed, amongst the top ten metabolites ranked by statistical significance in the old‐aged cluster at 72 years, seven metabolites (Table S3) were reported to significantly correlate with eGFR [31]. To further highlight the decline in renal function within individual age‐associated metabolic crests defined by DE‐SWAN, we manually selected ten metabolites implicated in CKD pathogenesis based on published literature [23, 31, 36], which included symmetric dimethylarginine, L‐kynurenine, citrulline, tryptophan 2‐C‐mannoside, succinyladenosine, pseudouridine, p‐cresol glucuronide, p‐cresol sulfate, creatinine, and uric acid. The number of CKD‐associated metabolites significantly altered in each of the three metabolic crests was compared, and the number of fluctuating CKD‐associated metabolites was evidently highest at old‐age (72 years), but statistical significance emerged as early as middle‐age (56 years) (Figure 1E,F).

### 2.3 Mapping Human Systemic Metabolite Patterns of Aging to Murine Organs and Tissues

**FIGURE 2:**
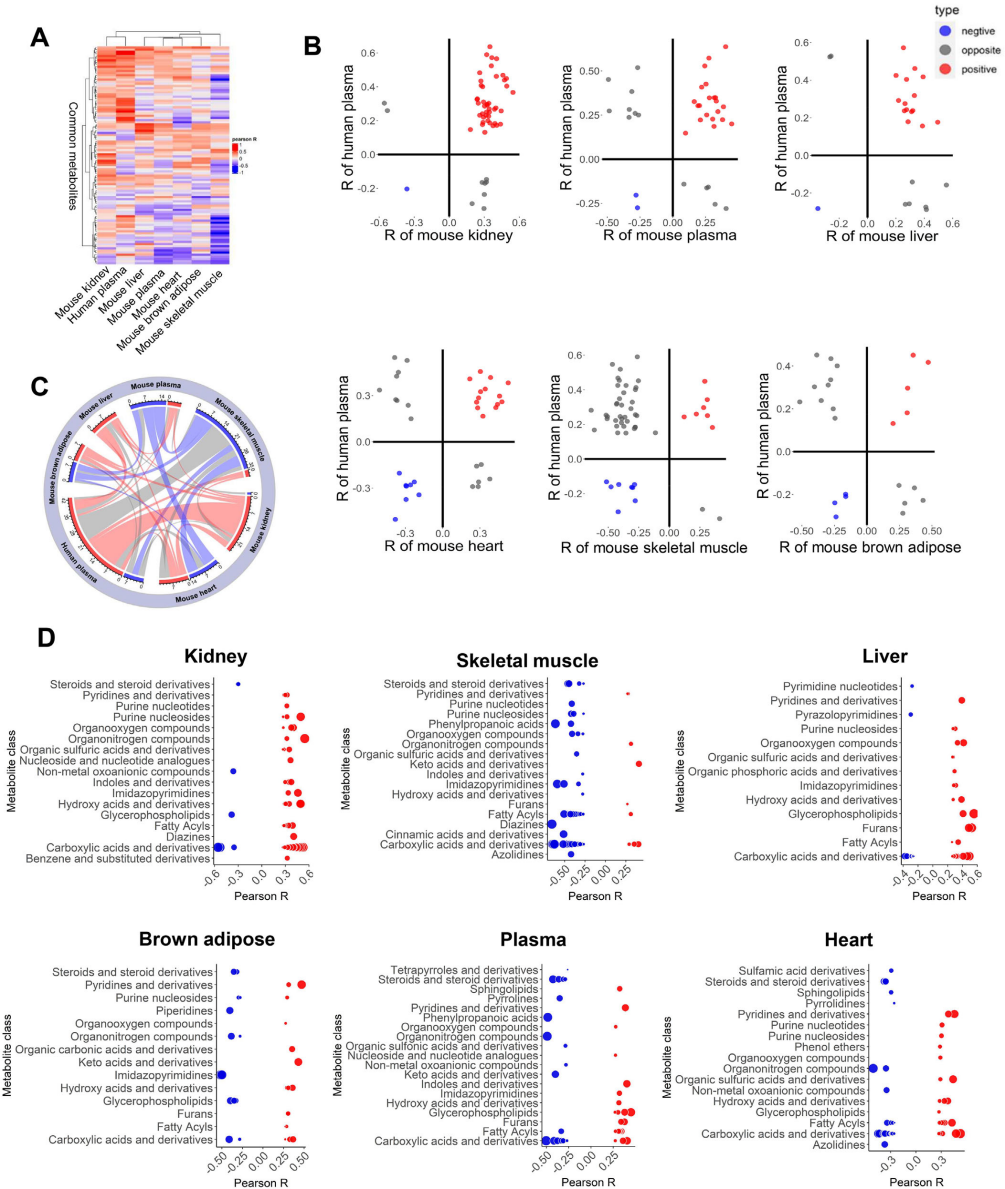
**Systemic changes of age‐associated metabolite patterns were mapped to the murine kidney**. (A) Heatmap with hierarchical clustering of commonly detected metabolites among mouse tissues and human plasma. Pearson correlation coefficients between metabolite levels and age were used to compare the Euclidean distance among individual mouse tissues and the human plasma. Positive correlations were indicated in red, and negative correlations were illustrated in blue. (B) Comparison of the age‐related metabolites in individual mouse tissues and the human plasma. Only metabolites significantly associated with age (*p* < 0.05) were illustrated. Red dots represent metabolites significantly and positively correlated with aging in both the mouse tissue specified and human plasma, while blue dots represent metabolites significantly and negatively correlated with aging. Gray dots denote metabolites significantly correlated with aging but with opposite directions of correlations between the mouse tissue specified and human plasma. Pearson method was used to calculate the correlation coefficients between metabolites and age, and statistically significant correlation was set at *p* < 0.05. (C) Chord diagram illustrates interorgan relationships in age‐associated metabolites. Age‐associated metabolites identified in murine organs/tissues, including the kidney, liver, plasma, skeletal muscle, brown adipose, and the heart, were compared with those in human plasma. The Pearson correlation method was used to calculate the correlation coefficients between metabolites and age, and a significant correlation was set at *p* < 0.05. For each pairwise comparison, metabolites positively correlated with age in both tissues/organs were categorized under the red band, and metabolites negatively correlated with age in both tissues/organs were grouped under the blue band. The width of the band indicates the number of significant correlations, and the color indicates the direction of correlations. Red and blue shades indicate consistently positive or negative correlations between two connecting tissues, respectively, while a gray shade indicates opposite directions of correlations. (D) Metabolite classes significantly correlated with aging in different mouse tissues. Sizes of circles were proportional to the magnitudes of the absolute Pearson correlation coefficients, and *p*‐value cut‐off was set at *p* < 0.1.

The foregoing results indicate significant overlap between the metabolite profiles of human systemic aging and renal dysfunction. To examine if the aging‐associated metabolite patterns in human is recapitulated in mice, we conducted in‐depth metabolomics analyses of five major peripheral organs and/or tissues in mice, including the kidney, skeletal muscle, liver, heart, brown adipose and plasma from 40 mice at 6 months (*n* = 5), 12 months (*n* = 12), 16 months (*n* = 12), and 20 months (*n* = 11) of age (Figure 2; Table S5), respectively, which corresponded approximately to the age span of our human cohort (20–88 years old). Pearson correlation was used to calculate the correlation coefficients of individual metabolites from the respective murine organs and/or tissues with age. Unsupervised hierarchical clustering showed that age‐associated metabolites from the human plasma and murine kidney were most similar, followed by murine liver and murine plasma (Figure 2A). Scatterplots of age‐associated metabolite correlations between human plasma and individual murine organs and/or tissues showed that relative to the human plasma, the murine kidney exhibited the greatest number of positively correlated age‐associated metabolites (red circles), while the skeletal muscle displayed the highest number of negative correlations (blue circles) (Figure 2B). A chord diagram was constructed for visualizing the directions of correlations across different tissues (Figure 2C). Corroborating preceding observations, consistently positive correlations (red bands) between human plasma and murine kidney, and opposite correlations (gray bands) between human plasma and murine skeletal muscles were observed (Figure 2C). Closer inspection of metabolite identities revealed that these human metabolite mediators of aging recapitulated in mice, were predominantly carboxylic acids and derivatives, which included fumaric acid, malic acid, creatine, creatinine, L‐cystine, and tryptophan 2‐C‐mannoside for murine kidney (Figure 2D). In addition, several age‐associated metabolites recapitulated in the mouse kidney are relevant to renal function (Table S6).

### 2.4 Murine Kidney GluCer Accumulation Underlies Age‐Associated Renal Functional Decline

**FIGURE 3:**
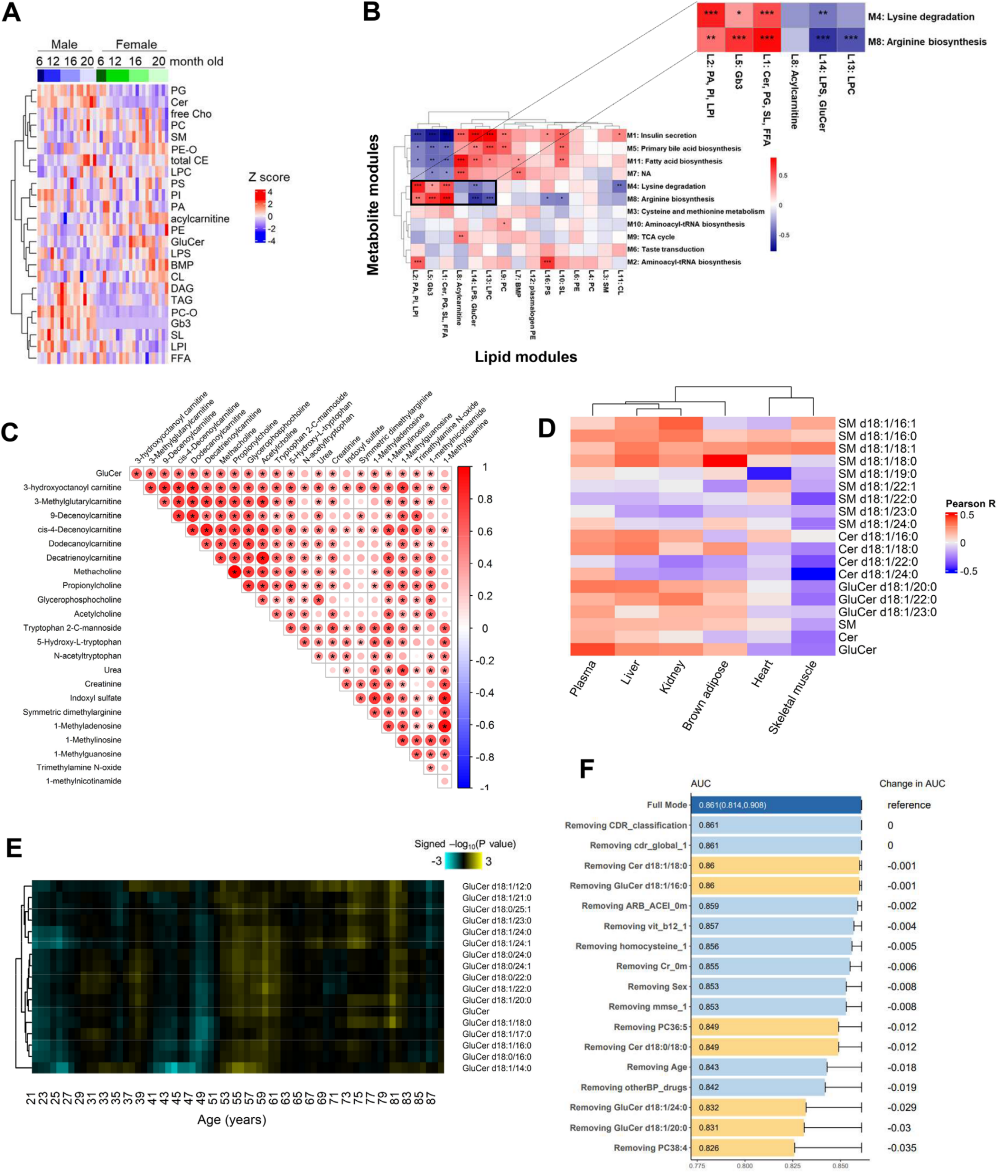
**Kidney GluCer accumulation underlies renal dysfunction across aging in the mouse**. A. Kidney lipidomic changes in male and female mice across aging. Kidneys collected from mice across four ages (6, 12, 16, and 20 months) were analyzed. Kidney lipid levels were z‐scored, and unsupervised hierarchical clustering aggregates lipid classes exhibiting similar temporal patterns of changes. (B) The heatmap illustrates the Pearson correlation between lipid and metabolite modules of the kidney from the WGCNA analysis. Expanded view on the upper panel displays metabolite modules (M4, M8) that were negatively correlated with lipid module L14 comprising GluCer, with functional characterization related to renal physiology. *p < 0.05; **p < 0.01; ***p < 0.001. Positive correlations were denoted in red and negative correlations in blue. (C) Triangular matrix displays correlations between total renal GluCer levels with endogenous abundances of metabolites implicated in renal function as measured in the kidney. Pearson's method was used to calculate the correlation coefficients; significant correlation was set at *p < 0.05. The size of the circles corresponds to the magnitude of the correlation coefficients. (D) Heatmap of correlations with age for sphingolipids commonly detected amongst murine tissues. Pearson correlation coefficients between metabolites and age were used to calculate the Euclidean distance among murine tissues. (E) Temporal waves of GluCer changes measured in human plasma characterized by DE‐SWAN (*n* = 222). Increases across the sliding age windows were indicated in yellow and decreases in turquoise. (F) Combinatorial panel of lipids and clinical indices associated with elevated risk of multiple causes of death in an elderly cohort. Area under the curve (95% confidence interval) was calculated using lasso regression (*n* = 271).

Our metabolomics‐oriented investigation underscores enhanced susceptibility of the kidney to aging‐induced functional decline. Aberrant lipid partitioning in CKD and ectopic renal lipid accumulation are known to undermine kidney function [37]. To elucidate molecular mechanisms underlying renal functional deficits with aging, we performed quantitative lipidomics on kidneys collected across four ages from both male (n = 19) and female (*n* = 23) mice (Figure 3A, Table S7). Changes in renal lipidomes across aging were sexually dimorphic (Figure 3A). Female‐specific accumulation of glucosylceramides (GluCer), acylcarnitines, cardiolipins (CLs), bis(monoacylglycero)phosphates (BMPs), and alkyl/alkenyl phosphatidylethanolamines (PE‐Os) were observed with increasing age, while age‐dependent accretions in neutral lipids, including diacylglycerols (DAGs), triacylglycerols (TAGs), and cholesteryl esters (CEs), were detected in male kidneys (Figure 3A). Weighted correlation network analysis (WGCNA) was applied for trans‐omics integration of renal metabolome and lipidome data, which were transformed into distinct metabolite and lipid modules (Tables S8–9). The strength of coregulation between individual metabolite and lipid modules was measured by Pearson correlation coefficients and represented in a clustered heatmap (Figure 3B). Metabolic signatures of individual metabolite modules were obtained by enriched pathways generated from overrepresentation analysis (ORA) of metabolites using the KEGG database, while lipid modules were defined by the predominant lipid classes within each cluster. We noticed prominent negative correlations between lipid module containing GluCer (L14) and metabolite modules denoting lysine degradation and arginine biosynthesis (Figure 3B, insert). The kidney represents the major site of arginine biosynthesis primarily via cells of the proximal convoluted tubules (PCTs) [38]. The kidney is also the principal organ responsible for the metabolic turnover of lysine, and accelerated lysine degradation is renoprotective under hypertension [39]. Integrated omics showed that GluCer accumulation in the kidney was associated with renal functional decline. A key feature of our chromatographic gradient is its ability to resolve GluCer from GalCer isomers [40], thus allowing for their unambiguous identification (Figure S2A). Total GluCer and individual GluCers with different fatty acyl chains began to accumulate in the kidneys of female mice at 16 months of age (Figure S2B‐C). Kidney GluCer levels were positively correlated with metabolites implicated in renal function and pathology, which include short‐ and medium‐chain acylcarnitines and N1‐methylated purines like 1‐methyladenosine and 1‐methylinosine (Figure 3C; Table S10). Amongst other organs, murine kidney and brain possess the highest levels of 1‐methyladenosine [41], and elevated circulating 1‐methyladenosine in CKD serves as an early indicator of cellular damage induced by oxidative stress [42].

### 2.5 Age‐Related Increases in Circulating GluCer Are Conserved in Human and Predictive of Death

Euclidean clustering using correlation coefficients between lipids and age revealed that the patterns of age‐associated sphingolipid changes in murine plasma were most similar to those in the liver and kidney (Figure 3D). Confining our analyses to only GluCer species commonly detected in the plasma and individual organs/tissues, the shortest Euclidean distance was again measured between plasma and the kidney (Table S11). These results highlight the kidney as the major peripheral organ associated with age‐dependent changes in circulating GluCer. DE‐SWAN analysis of quantified GluCer levels in plasma samples from the cross‐sectional aging cohort (Table 1) uncovered a small wave of GluCer increases at 54–55 years (Figure 3E). Circulating GluCers exhibited increasing trends with age (Figure S2D; Tables S12 and S13), which were particularly significant in females (Figure S2E). DE‐SWAN analysis revealed a crest in circulating GluCer levels of females at 54–61 years following a trough from 47 to 51 years, while a small crest at 71–72 years was observed in males (Figure S3A). Total plasma GluCer was significantly and positively correlated with age for females, but not for males (Figure S3B,C). Of interest, DE‐SWAN analysis uncovered waves of increases in circulating uremic toxins at 51 and 71 years for females, and at 71 years for males (Figure S3D), which closely mirrored temporal waves of increases in circulating GluCer. A closer look at the age‐dependent variations in major uremic toxins, including uric acid, creatinine, and L‐Kynurenine, showed that females generally maintained considerably lower levels of these uremic toxins relative to males up to about 50 years old, following which the levels of uremic toxins increased steadily to comparable levels in males after 60 years old (Figure S3E). Thus, the rise in plasma uremic toxins closely coincided with temporal crests of GluCer accretion throughout the life course of females. In a separate longitudinal cohort of elderly people (*n* = 271) (Table 2), baseline levels of circulating GluCer d18:1/20:0 and GluCer d18:1/24:0, together with PC38:4, were identified as the top three variables predictive of deaths within a follow‐up period of 6 years (Figure 3F). The combinatorial panel of circulating lipids and clinical indices measured at baseline was predictive of deaths predominantly resulting from organ failure, infection, or cardiovascular events, with an area under the curve (AUC) of 0.861 (95% CI = 0.814–0.908) using lasso regression analysis (Figure S3F,G). Therefore, age‐associated increases in circulating GluCer, largely contributed by the kidneys, are conserved from mice to humans, and are significantly associated with enhanced risk of multiple causes of mortality in aged individuals.

**TABLE 2:** Clinical characteristics of longitudinal cohort of elderly people.

Characteristic	Deaths, *N* = 94	Survivors, *N* = 177	*p*‐value
Age (years)			<0.001^a^
Mean (SD)	76.44 (4.29)	74.54 (3.75)	
Min–max	69.00–85.00	69.00–85.00	
Median (Q1,Q3)	76.00 (73.00,80.00)	74.00 (71.00,77.00)	
Sex, *n* (%)			0.109^b^
Male	61 (65%)	97 (55%)	
Female	33 (35%)	80 (45%)	
Smoking, *n* (%)			0.076^b^
Nonsmoker	42 (56%)	102 (66%)	
Smoker	10 (13%)	8 (5.2%)	
Ex‐smoker	23 (31%)	44 (29%)	
Unknown	19	23	
BMI, kg/m^2^			0.443^a^
Mean (SD)	25.23 (3.48)	24.89 (3.05)	
Min–max	16.08–36.39	17.89–32.18	
Median (Q1, Q3)	24.85 (22.89, 27.61)	24.92 (22.91,27.03)	
Unknown	5	6	
eGFR, mL/min/1.73 m^2^			<0.001^a^
Mean (SD)	62.43 (18.25)	72.04 (19.25)	
Min–max	28.69–112.98	26.05–131.18	
Median (Q1, Q3)	59.80 (50.19, 73.36)	71.37 (58.33, 84.75)	

Abbreviation: eGFR: estimated glomerular filtration rate.

^a^ Welch Two sample *t*‐test.

^b^ Pearson's Chi‐squared test.

### 2.6 Trans‐Omics Elucidation on the Function of Circulating GluCer Across Aging

**FIGURE 4:**
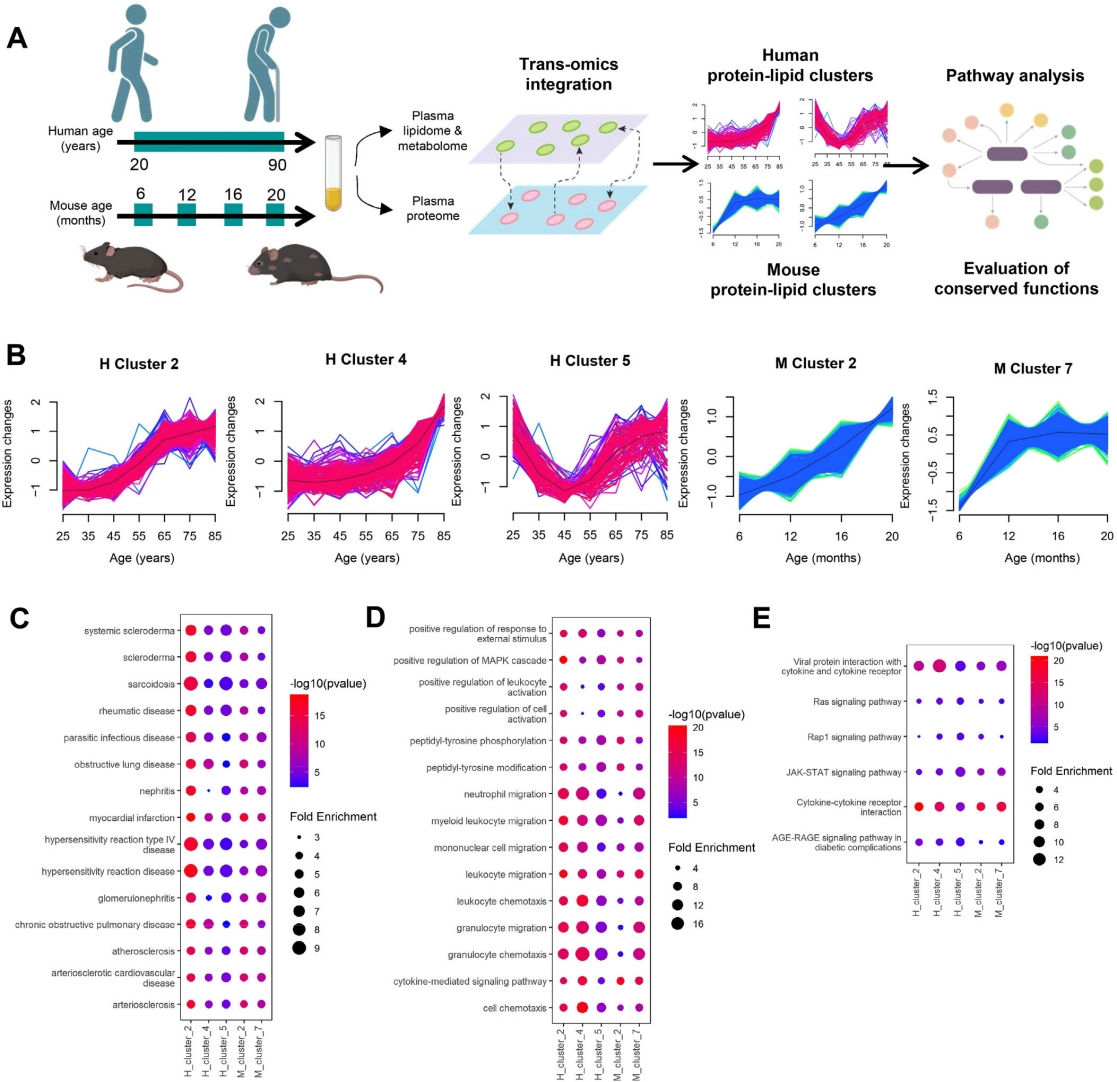
**Trans‐omics evaluation of systemic GluCer function conserved between humans and mice**. (A) Trans‐omics data integration of plasma proteome and lipidome aging datasets to elucidate functional pathways associated with circulating GluCer levels conserved in human and mice. (B) Clusters of lipid–protein clusters across aging that contained GluCer species from human (H cluster2, H cluster 4, and H cluster 5) and mice (M cluster 2 and M cluster 7). (C) Disease Ontology (DO) enrichment of the five lipid–protein clusters containing GluCer. Enrichment was tested using the hypergeometric test. (D) Gene Ontology (GO) biological process enrichment of the five lipid–protein clusters containing GluCer. Enrichment was tested using the hypergeometric test. (E) KEGG pathway enrichment of the five lipid–protein clusters containing GluCer. The size of circles represents the magnitude of fold enrichment, while the magnitudes of *p*‐values are indicated by the color bar. Enrichment was tested using the hypergeometric test.

Using published datasets of human and mouse plasma proteomes across aging [33], we conducted trans‐omics integration of the senescence‐associated plasma proteome with metabolome and lipidome obtained in our study. Distinct human and murine protein‐lipid clusters were captured (Tables S14 and S15), which comprised proteins and lipids/metabolites exhibiting comparable temporal patterns across aging. ORA analysis of enriched proteins within individual clusters was performed against the Disease Ontology (DO), Gene Ontology (GO), and KEGG pathway database to define the biological function of these senescence dynamic clusters (Figure 4A). Individual GluCer species were distributed amongst H_cluster 2, H_cluster 4, and H_cluster 5 of the human protein–lipid clusters, and M_cluster 2 and M_cluster 7 of the murine protein–lipid clusters, respectively. H_cluster 2 and M_cluster 7 possessed very similar temporal patterns across aging, with sharp increases particularly from young through the middle age, and attenuated increases in old age. Both clusters comprised GluCer d18:0/22:0, GluCer d18:0/24:1, GluCer d18:0/24:0, and GluCer d18:1/22:0 that were predominantly dihydro‐GluCer species containing very long‐chain fatty acyls (≥C22) (Figure 4B). The matching temporal patterns of changes in these GluCer across senescence suggest that these lipids might have conserved functions between humans and mice that are relevant to systemic aging. Top 15 DO terms ranked by statistical significance conserved across these five GluCer‐associated clusters implicated several peripheral organs and tissues, including the kidney (nephritis, glomerulonephritis), heart (myocardial infarction), and lungs (obstructive lung disease, chronic obstructive pulmonary disease) (Figure 4C), which suggests that circulating GluCer accumulation might impact multiple organs across aging. With regard to GO terms, “neutrophil migration” and “granulocyte migration and chemotaxis” were the most significantly enriched terms across the five GluCer‐associated clusters (Figure 4D). The “cytokine–cytokine receptor interaction”, “JAK‐STAT signaling pathway”, and “AGE‐RAGE signaling pathway in diabetic complications” emerged as the most significant pathways associated with these GluCer clusters from ORA analysis using the KEGG pathway database (Figure 4E; Figure S4).

### 2.7 Mitochondria Activity of PCTCs From Older Mice Is Sensitive to GluCer Accumulation

**FIGURE 5:**
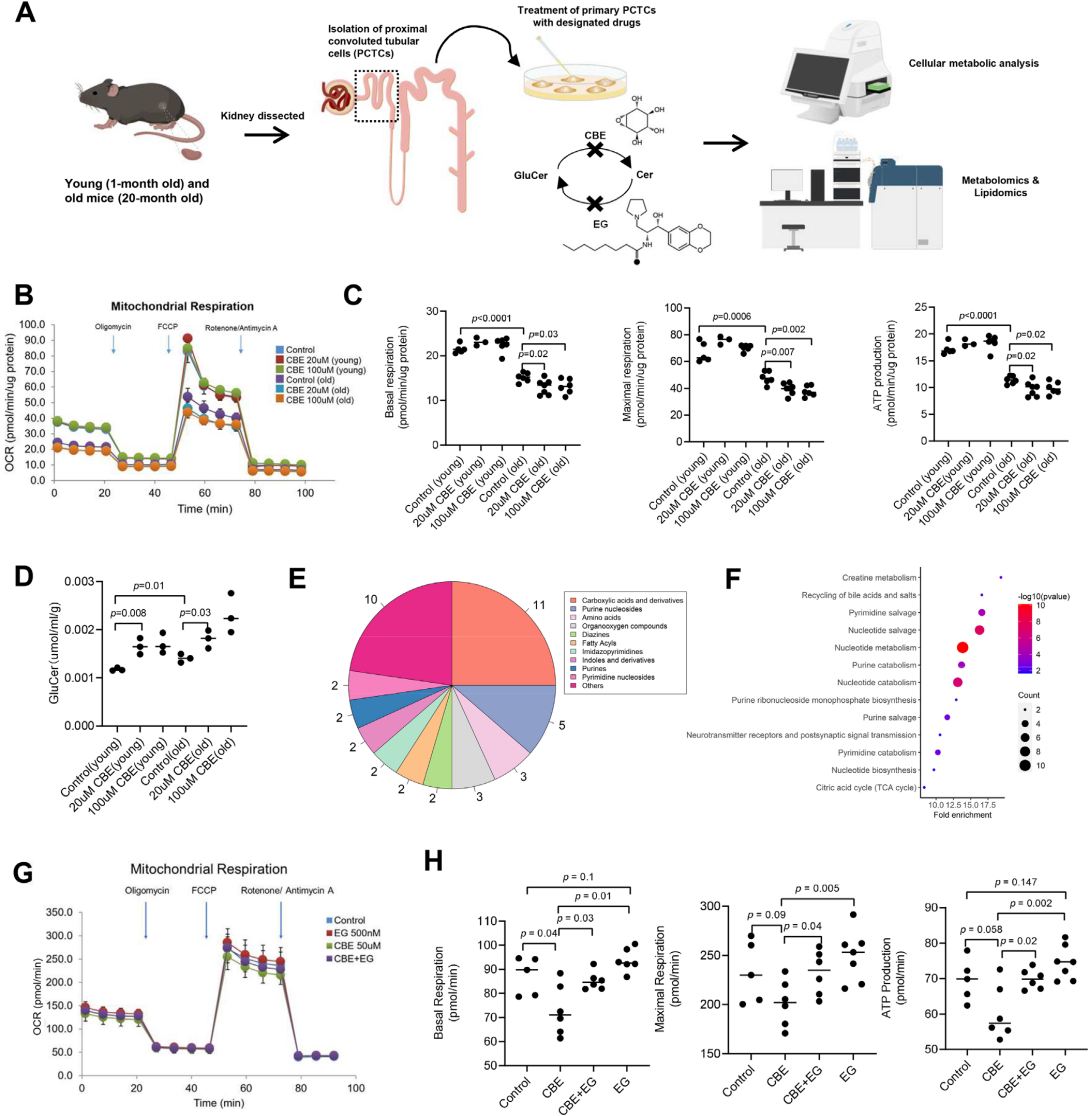
**Mitochondria isolated from PCTCs of old mice are sensitive to endogenous GluCer accumulation**. (A) PCTCs were isolated from kidneys dissected from young (1‐month old, *n* = 4) and old (20‐month‐old, *n* = 4) mice for mitochondrial respiration and function assays. (B) Oxygen consumption rates (OCR) of PCTCs isolated from young and old mice treated with control saline or CBE (20 or 100 µM) were measured by the Seahorse XeF96 extracellular flux analyzer. Error bars represent SEM. (C) Mitochondrial basal respiration, maximal respiration and ATP production following treatments with CBE, the groups are namely: Control (young; *n* = 5), CBE 20 µM (young; *n* = 3), CBE 100 µM (young; *n* = 6), Control (old; *n* = 6), CBE 20 µM (old; *n* = 7), and CBE 100 µM (old; *n* = 6). Statistical significance was determined using a two‐tailed unpaired *t*‐test. (D) GluCer content of mouse PCTCs (obtained from young or old mice) treated with different concentrations of CBE (20 or 100 µM) for 48 h. Statistical significance was determined using a two‐tailed unpaired *t*‐test. (E) Major metabolite classes were elevated in 20‐month‐old mice (*p* < 0.1) after 100 µM CBE treatment compared with control mice. (F) Pathways significantly enriched (*p* < 0.05) for metabolites elevated in 20‐month‐old mice with 100 µM CBE treatment compared with control mice (*p* < 0.1). Enrichment was tested using a hypergeometric test based on the Reactome database; the *p*‐value cut‐off was set at *p* < 0.05. (G) Mitochondrial respiration of PCTCs as indicated by oxygen consumption rate (OCR). Error bars represent s.e.m. CBE + EG group denotes treatment with 50 µM CBE + 500 nM EG. (H) Mitochondrial basal respiration, maximal respiration, and ATP production of PCTCs from 20‐month‐old male mice after treatments with different concentrations of CBE and EG. The groups included control (*n* = 5), CBE 50 µM (*n* = 6), 50 µM CBE + 500 nM EG (*n* = 6), and EG 500 nM (*n* = 6). *p*‐values presented were determined using a two‐tailed unpaired *t*‐test. PCTCs: proximal convoluted tubule cells; CBE: conduritol‐β‐epoxide; EG: Eliglustat.

Biosynthesis of GluCer involves the addition of a glucose moiety to Cer precursors mediated by UDP‐glucose ceramide glucosyltransferase (UGCG) in the Golgi apparatus [43], while glucocerebrosidase/glucosylceramidase (GCase) is a lysosomal enzyme that breaks down GluCer into glucose and Cer constituents. GCase is encoded by the glucosylceramidase beta (*GBA*) gene located on chromosome 1q21. To further explore the biological effect of GluCer accumulation on renal metabolism and uremic toxin production, PCT cells (PCTCs) were isolated from kidneys dissected from young (1‐month‐old) and old (20‐month‐old) mice and treated with 20 or 100 µM of conduritol‐β‐epoxide (CBE) (Figure 5A). CBE covalently and irreversibly binds to the catalytic site of GCase, thereby leading to irreversible inactivation of the enzyme and subsequent GluCer accumulation [44]. PCTCs were selected for assay because the proximal tubule displays the most prominent sex‐biased molecular programs in the kidney and undergoes strong, sex‐dependent remodeling with aging, while also serving as a primary site for renal elimination of diverse exogenous and endogenous toxins (e.g., uremic toxins) [45]. Oxygen consumption rates (OCR) of PCTCs isolated from young and old mice treated with control saline or CBE (20 or 100 µM) were measured by the Seahorse XeF96 extracellular flux analyzer, and the basal respiration, maximal respiration, and ATP production from these cells were calculated (Figure 5B,C). The oxygen consumption rates of PCTCs isolated from young mice were unaffected by CBE, both at 20 and 100 µM, indicating that these concentrations of CBE were not physiologically toxic to the mitochondria of young PCTCs. The OCR, respiratory rates, and ATP production of PCTCs from old mice, however, were significantly decreased with CBE treatment relative to the control group. In addition, CBE treatment at both concentrations led to significantly elevated GluCer levels in PCTCs from both young and old mice (Figure 5D). Also, in accordance with our preceding observations, GluCer levels were higher in untreated PCTCs from old compared with young control mice (Figure 5D). In addition to our LC/MS‐based quantification of GluCer (Figure S2A), we further validated its accumulation using an anti‐GluCer antibody, which specifically recognizes GluCer over GalCer, for immunocytochemical staining of primary PCTCs isolated from female mice. Immunocytochemistry results supported LC/MS data indicating specific and significant accumulation of GluCer in PCTCs with CBE treatment, particularly for PCTCs isolated from old mice (Figure S5A). The foregoing data indicate that while mitochondria of young PCTCs can accommodate elevated levels of GluCer resulting from impeded hydrolysis, mitochondria in PCTCs of old mice are sensitive to GluCer accretion, as shown by compromised oxidative phosphorylation and ATP production. Metabolomics revealed that PCTCs of old mice treated with 100 µM CBE were significantly elevated in numerous metabolite classes relative to untreated controls, particularly for carboxylic acids and derivatives and purine nucleotides (Figure 5E). Several of these metabolites, such as pseudouridine, symmetric dimethylarginine, 1‐methylguanosine, L‐kynurenine, 1‐methyladenosine, and other uremic toxins, are known metabolite markers of CKD (Table S16). Reactome pathways involving nucleotide metabolism and nucleotide salvage denote the most significantly altered metabolic pathways following CBE treatment at 100 µM relative to control saline (Figure 5F).

To demonstrate that abrogating GluCer accumulation can restore mitochondrial function in PCTCs, we utilized Eliglustat (EG), a specific and potent inhibitor of GluCer synthase that is currently used for the treatment of Gaucher's disease. As it was shown that only PCTC mitochondria from old mice are sensitive to GluCer increase (Figure 5B,C), we utilized PCTCs isolated from old mice for subsequent cell‐based experiments. Combinatorial treatment with CBE (50 µM) and EG (500 nM) restored the OCR of PCTCs from 20‐month‐old mice to levels comparable to the control group (Figure 5G). Conforming with expectations, EG enhanced maximal respiration of PCTCs relative to control, and addition of EG to CBE‐treated PCTCs significantly improved basal respiration, maximal respiration, and enhanced ATP production compared with CBE‐treated cells (Figure 5H). EG treatment also reversed the metabolome profiles of PCTCs relative to CBE treatment. For example, pseudouridine, creatine, tryptophan 2‐C‐mannoside, and other uremic toxins, which were elevated in CBE‐treated PCTCs, were reduced in PCTCs treated with EG (Table S17).

### 2.8 Age‐Associated Vulnerability of Renal Mitochondria Clearance to Glycosphingolipid Imbalance

**FIGURE 6:**
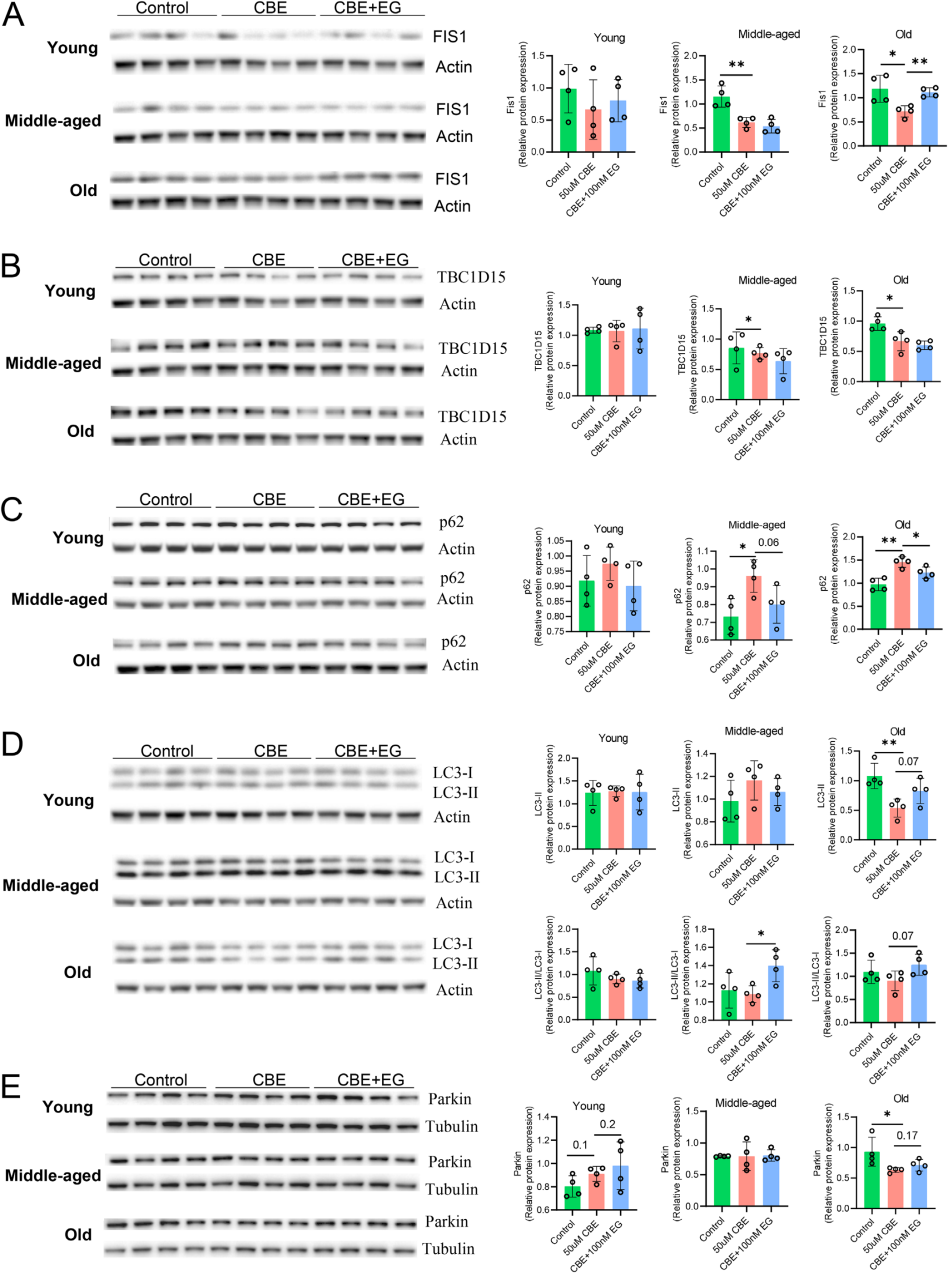
**Age‐dependent effects of CBE on protein expression in primary mouse PCTCs**. (A–E) Primary PCTCs isolated from young (5–6 months), middle‐aged (14–16 months), and old (23–24 months) female mice were treated with 50 µM CBE with or without EG‐mediated glucosylceramide synthesis inhibition. Protein levels were assessed by immunoblotting. Barplots on the relative protein expressions of Fis1, TBC1D15, p62, LC3, and Parkin. Data are presented as means ± SD. One‐way ANOVA followed by Dunnett's post hoc test versus the CBE group. *p < 0.05, **p < 0.01.

Analysis of PCTCs isolated from young (5–6 months), middle‐aged (14–16 months), and old (23–24 months) female mice revealed distinct age‐dependent responses to 50 µM CBE. CBE exposure significantly elevated GluCer levels across all age groups, an effect that was ameliorated by EG treatment (Figure S5B). We observed pronounced age‐related differences in markers of mitochondrial quality control and autophagy. CBE treatment significantly reduced FIS1 protein levels in middle‐aged and old PCTCs, but not in young cells; this loss was restored by EG co‐treatment specifically in the old group (Figure 6A). Similarly, TBC1D15 expression—a regulator of mitochondria–lysosome (M–L) contacts that promotes mitochondrial quality control through mitophagy [46] was significantly downregulated by CBE in middle‐aged and old groups, but remained unaltered in young PCTCs (Figure 6B). CBE treatment also impairs autophagy in an age‐dependent manner. The autophagy adaptor p62 significantly accumulated in CBE‐treated middle‐aged and old PCTCs, but was unaffected in young cells, and EG treatment significantly attenuated such accumulations (Figure 6C). Furthermore, CBE selectively reduced LC3B‐II levels in the old group, an effect reversed by EG. Notably, EG also elevated the LC3B‐II/LC3B‐I ratio in middle‐aged and old groups (Figure 6D), suggesting enhanced autophagic activation by ameliorating GluCer accumulation. Parkin expression decreased significantly in CBE‐treated old PCTCs, with EG showing a restorative trend (*p* = 0.17), while no change was observed in young and middle‐aged groups (Figure 6E).

**FIGURE 7:**
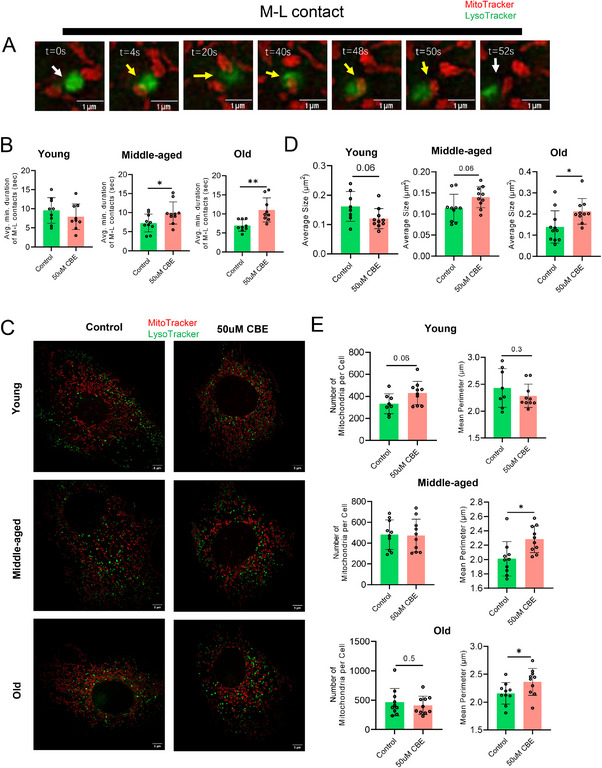
**Age‐dependent alterations in mitochondria–lysosome contact dynamics and organellar morphology induced by CBE treatment**. (A) Representative time‐lapse confocal images illustrating mitochondrial (red) and lysosomal (green) interaction dynamics in PCTCs, capturing key stages of organelle tethering and untethering. Yellow arrows indicate mitochondria–lysosome (M–L) contact sites (*t* = 0–52 s). (B) Quantification of M–L contact duration. (C) Confocal micrographs of mitochondria (red) and lysosomes (green) in PCTCs isolated from young (5–6 months), middle‐aged (14–16 months), and old (23–24 months) female mice under control and CBE‐treated conditions. (D) Lysosomal size analysis. (E) Mitochondrial morphology parameters: mitochondria count, mean perimeter, mean aspect ratio, and mean branch length. Data are presented as means ± SD. Statistical significance was determined using a two‐tailed unpaired *t*‐test. *p < 0.05, **p < 0.01.

To functionally assess interorganellar dynamics, we performed time‐lapse super‐resolution live imaging (HIS‐SIM) of M–L contacts in PCTCs from young (5–6 months), middle‐aged (14–16 months), and old (23–24 months) female mice. A 48 h treatment with 50 µM CBE significantly prolonged the average duration of M–L contacts exclusively in middle‐aged and old groups compared with age‐matched controls (Figure 7A,B), consistent with prior observations in neuronal models where GluCer accumulation disrupts M–L untethering dynamics [46]. Furthermore, CBE induced a marked increase in lysosomal size specifically in old PCTCs (Figure 7C,D). Analysis of mitochondrial morphology revealed that mitochondrial numbers were unaltered with CBE treatment across the three age groups (Figure 7C,E). However, CBE significantly increased the mean mitochondrial perimeter in both middle‐aged and aged groups, but not in young PCTCs (Figure 7E)—indicative of aberrant mitochondria elongation and fragmentation. To summarize, age‐dependent GluCer accumulation, recapitulated herein by CBE treatment, triggers mitochondrial dysfunction in PCTCs via dysregulated fission (reduced FIS1), disrupted interorganellar dynamics (prolonged M–L contacts), and impaired autophagic clearance (p62 accumulation, reduced LC3B‐II). These observations reveal that GluCer accumulation preferentially disrupts mitochondria dynamics and interorganellar coordination underlying autophagic clearance of damaged mitochondria in old PCTCs, highlighting an age‐associated mitochondria vulnerability to renal glycosphingolipid imbalance.

### 2.9 Resiliency Mechanisms of Late Middle‐Aged Females in Coping with Renal GluCer Accumulation

As sharp increases in circulating uremic toxins were observed in human females at the late middle‐age stage (approximately 50–60 years) (Figure S3E), we created a murine model to investigate resiliency mechanisms that might operate in the middle‐aged to maintain renal function despite GluCer accumulation. A murine model of GluCer accumulation was established by intraperitoneal injection of young (4‐month‐old) and middle‐aged (14‐month‐old) female mice with CBE (100 mg/kg/day) for a period of 21 days, and the control group underwent saline injection (Figure S6A). Late middle‐aged mice exhibited close to 40% reduction in body weight at 21 days with CBE injection, while no significant changes in body weight were noted for young mice (Figure S6B). CBE injection resulted in significant increases in kidney GluCer levels in both young and late middle‐aged mice (Figure S6C), but pathological changes in renal tubules were observed only in late middle‐aged mice (Figure S6D‐E). Hematoxylin–eosin staining showed that the renal tubule morphology of CBE‐injected mice was more irregular, with ectopic lipid accumulation represented by white unstained areas (Figure S6D). The ratio of unstained areas to total surface areas of renal tubules was calculated to reflect the degree of renal tubule damage, which was elevated in CBE‐treated mice relative to control mice (Figure S6E). Metabolomic profiling revealed that elevated long‐chain acylcarnitines, creatine, and creatinine, alongside reductions in nucleotides such as uridine, guanosine, and hypoxanthine, were reduced in CBE‐treated late middle‐aged mice (Figure S6F; Table S18). Reactome pathway analysis indicated upregulation of compensatory metabolic processes, including SLC‐mediated transmembrane transport of cations/anions, amino acids and organic acids, the citric acid cycle and respiratory electron transport, as well as purine catabolism (Figure S6G). Specifically, metabolic products of purine catabolism (xanthosine and uric acid) were increased with CBE treatment, while adenosine and guanosine were reduced (Table S18). We propose that these metabolic adaptations represent resiliency mechanisms that help maintain renal function in late middle‐aged female mice despite GluCer accumulation, and before the onset of overt uremia.

At the molecular level, CBE‐induced GluCer accumulation disrupted mitochondria dynamics in late middle‐aged female kidneys by skewing toward fission (increased Drp1 and reduced Opa1) (Figure S7A–C). We also observed activation of the mammalian target of rapamycin complex 1 (mTORC1) pathway (elevated p‐mTOR/mTOR and p‐S6K/S6K ratios) (Figure S7G,H), which is known to suppress mitophagy [47]. Despite these changes, renal mitochondria of late middle‐aged females largely remained polarized and functional, as indicated by unaltered levels of PTEN‐induced kinase 1 (Pink1) (Figure S7D)—a marker of mitochondrial depolarization [48], and no induction of cellular senescence (unchanged p53 levels) (Figure S7E). Together, our findings indicate coordinated but insufficient metabolic adaptations, such as enhanced purine catabolism and upregulation of energetic pathways, to preserve renal mitochondrial function despite accrual of tubular damage at late middle‐age.

### 2.10 Depletion of Endogenous Purines Mitigates GluCer‐induced mTOR Signaling Dysregulation and Mitochondrial Dysfunction

**FIGURE 8:**
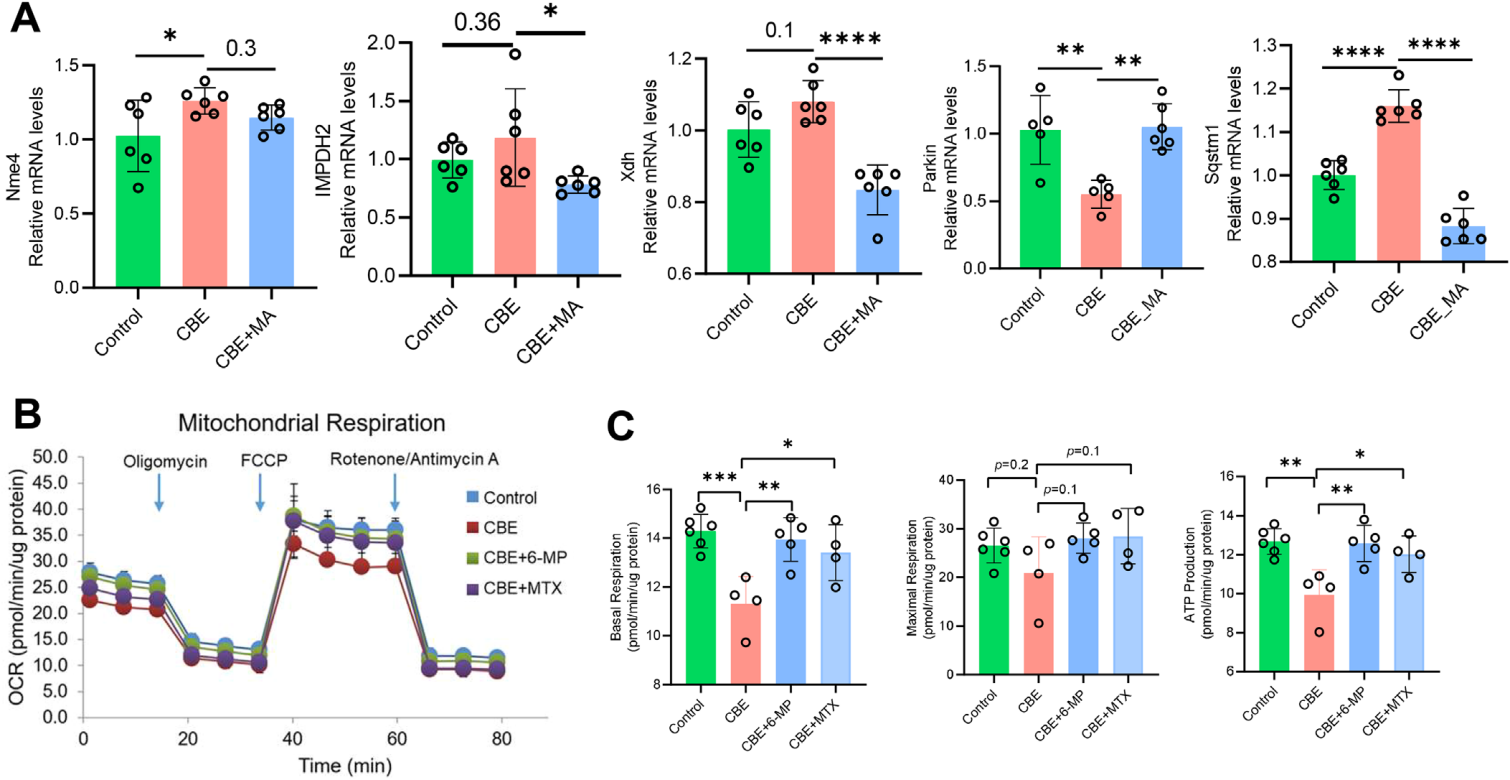
**CBE‐induced dysregulation of mTOR signaling and mitochondrial dysfunction was mitigated by depletion of endogenous purines in vitro**. (A) Primary PCTCs isolated from 23‐month‐old female mice were treated with vehicle (Control), 50 µM CBE, or 50 µM CBE + 2 µM MA for 48 h. Relative mRNA expressions were measured by qPCR (*n* = 6). (B) Mitochondrial basal respiration, maximal respiration, and ATP production of PCTCs from 16‐month‐old female mice under different treatments, including control (*n* = 6), 20 µM CBE (*n* = 4), 20 µM CBE + 20 µM 6‐MP (*n* = 5), and 20 µM CBE + 2 µM MTX (*n* = 4). (C) Mitochondrial respiration of PCTCs, as indicated by oxygen consumption rate (OCR). CBE refers to treatment with 20 µM CBE; CBE + 6‐MP group denotes treatment with 20 µM CBE + 20 µM 6‐MP; CBE+MTX group denotes treatment with 20 µM CBE+ 2 µM. Data are presented as means ± SD. One‐way ANOVA followed by Dunnett's post hoc test versus the CBE group. *p < 0.05, **p < 0.01, ***p < 0.001, ****p < 0.0001. CBE: conduritol‐β‐epoxide; MA: mycophenolic acid; MTX: methotrexate (MTX); 6‐MP: 6‐mercaptopurine; PCTCs: proximal convoluted tubule cells.

Given that purine catabolism may represent a resiliency mechanism against GluCer‐induced renal functional decline, we investigated the role of endogenous purines using mycophenolic acid (MA), an inhibitor of de novo purine biosynthesis, in PCTCs isolated from 23‐month‐old female mice. Cells were treated for 48 h with 50 µM CBE alone or co‐treated with 50 µM CBE and 2 µM MA (CBE+MA). qPCR analysis showed that CBE perturbed purine metabolism by significantly upregulating *Nme4*, a nucleoside diphosphate kinase that promotes nucleoside triphosphate synthesis. While MA co‐treatment did not alter *Nme4* expression, it reduced mRNA levels of *Impdh2* (key enzyme in *de novo* purine biosynthesis) and *Xdh* (involved in purine breakdown) (Figure 8A), indicating an overall reduction in purine production and metabolism. Notably, MA co‐treatment reversed the CBE‐induced reduction in *Parkin* expression and increase in *Sqstm1* (which encodes the autophagy adaptor p62) (Figure 8A), consistent with our earlier immunoblot results in aged PCTCs (Figure 6). These in vitro results support our hypothesis that purine accumulation drives aberrant mitophagy under conditions of GluCer overload, corroborating a prior report that intracellular purines modulate mTORC1 activation [49].

We next investigated whether inhibiting purine biosynthesis or salvage could directly rescue mitochondrial function. Using Seahorse metabolic flux analysis, we treated PCTCs for 24 h with CBE alone or in combination with inhibitors of purine production: methotrexate (MTX, a dihydrofolate reductase [DHFR] inhibitor that blocks de novo purine synthesis) or 6‐mercaptopurine (6‐MP, a hypoxanthine‐guanine phosphoribosyltransferase [HPRT1] that blocks purine salvage). Strikingly, co‐treatment with either 6‐MP or MTX restored OCR levels in CBE‐challenged PCTCs to those of saline‐treated controls (Figure 8B,C). Deficits in both basal respiration and ATP production in CBE‐treated PCTCs from aged mice were fully rescued by concurrent inhibition of purine synthesis or salvaging (Figure 8B,C). Together, these results demonstrate that the adverse effects of GluCer accumulation on mitochondrial metabolism in aged PCTCs are mediated by purine‐dependent mTORC1 activation and can be ameliorated by depletion of endogenous purines.

### 2.11 Upstream GluCer Handling Accounts for Sexual Dimorphism in Renal Vulnerability to Aging

To determine if the GluCer‐purine‐mTORC1 axis is conserved in male mice, we artificially induced renal GluCer accumulation in male mice—which do not naturally accrue GluCer in kidneys with age (Figure 3A)—via a 35‐day treatment regimen (Figure S8). CBE administration (100 mg/kg) was halted on day 28, while the mice receiving MA (100 mg/kg) co‐treatment to deplete purines (CBE+MA) continued receiving MA for an additional seven days before mice sacrifice and sample collection. CBE treatment induced significant mortality by day 31 (3/9), which was completely prevented by co‐treatment with MA (Figure S8A). Masson's Trichrome staining revealed that CBE induced borderline renal fibrosis that was attenuated by MA co‐administration (Figure S8B). Notably, CBE challenge in males recapitulated the pathogenic cascade observed in females, which encompassed activation of mTOR signaling (decreased *PRAS40*, increased *Rictor*, elevated p‐mTOR/mTOR protein ratios) (Figure S8C,D), induction of a pro‐fission shift (reduced *MFN1* and *MFN2*) with impaired mitochondrial biogenesis (reduced *Ppargc1a*) and dysregulated mitophagy by downregulation of both *Parkin* and the M–L tethering gene *TBC1D15*. Crucially, MA co‐treatment significantly ameliorated these molecular defects, particularly in restoring the expressions of genes governing mTOR regulation (*Deptor* and *Rictor*), mitochondrial fusion (*MFN1*), and mitophagy (*Ppargc1a, TBC1D15*) (Figure S8C), while showing a strong tendency toward reducing mTOR phosphorylation (Figure S8D). Collectively, these data demonstrate that the core GluCer‐purine‐mTORC1‐mitophagy pathway is conserved in males, and that female‐specific vulnerability to age‐related renal decline is ascribed to sexually dimorphic upstream handling of glycosphingolipids, leading to preferential GluCer accumulation in aging females that drives this conserved pathological cascade.

## 3 Discussion

Despite being a central hub of mammalian metabolism, the metabolic importance of the kidney in modulating systemic aging is relatively understudied. The kidney devotes high energy expenditure toward maintaining electrolyte and fluid homeostasis, and in ensuring efficient waste removal and nutrient uptake, making it particularly susceptible to age‐induced metabolic disturbances [50]. Our metabolism‐focused approach unraveled enhanced vulnerability of the kidney to age‐related functional decline, as evident by the predominance of metabolites associated with renal function in the old‐aged cluster at 72 years from DE‐SWAN analysis. Cross‐tissue/organ correlation analyses unveiled that a majority of the aging characteristics observed in the human plasma were most strongly associated with age‐associated changes in the kidney. Deteriorated renal function induces an augmented release of uremic toxins into the circulation, which serve as systemic metabolite mediators triggering aberrant interorgan crosstalk and multiple organ dysfunction [51], thereby accelerating systemic aging. Previous studies revealed that the decline in kidney function increases the risk of developing cardiovascular diseases by two‐ to fourfold [52], with advanced stage (stage 3–5) CKD patients having significantly elevated risk [53].

Through integrating metabolomics and lipidomics data, we discovered sexually dimorphic alterations in the kidney lipidome across aging. In particular, renal accumulation of GluCer was evident specifically in females from the late middle‐age phase onwards, and this female‐specific accrual of GluCer at late middle‐age was conserved across humans and mice. Importantly, late middle‐age onset accumulation of GluCer closely mirrors the surge in circulating uremic toxins in human females between 50 and 60 years old. While specific uremic toxins might stem from nonrenal sources, for example, gut‐derived metabolites, including p‐cresol sulfate and p‐cresol glucuronide, our reported panel of age‐associated uremic toxins comprised predominantly host‐derived metabolites that accumulate as renal function declines. These observations point to a plausible link between renal GluCer accumulation and decline in kidney function, reflected by increased circulating uremic toxins, specifically in females. In this regard, tissue GluCer has been implicated as a determinant of organ size, and enzymes controlling GluCer levels (i.e., UGCG and GCase) were demonstrated to be under hormonal control. Testosterone was shown to increase UGCG activity while reducing GCase activity, giving rise to enhanced GluCer levels and rapid kidney growth. On the other hand, estradiol was found to attenuate kidney growth by increasing GCase activity and suppressing UGCG activity in mice [54]. We postulate that the female‐specific accretion in kidney GluCer observed at the late middle‐age may be ascribed to a decline in estradiol level with aging. Indeed, the rise in GluCer levels coincided with the age range of menopause onset for human females (ca. 45–55 years), and also corresponded to a period of drastic estradiol reductions measured in female mice between 12 and 17 months of age [55].

We then explored the systemic effects of senescence‐associated GluCer accumulation by combinatorial analyses of plasma lipidomics and proteomics data across aging in humans and mice. We emphasized lipid–protein modules with conserved functions between the two species, which underscored the role of circulating GluCer in modulating the migration and chemotaxis of immune cells. Increased production of proinflammatory cytokines, including various interleukins and chemokines, had been previously reported for human and murine macrophages challenged with GluCer overload, and immune cells of Gaucher's disease patients are “primed” for facilitated release of cytokines [56, 57]. In a similar light, elevated circulating GluCer during senescence may promote cytokine release following immune cell uptake, thereby skewing systemic immune profiles toward the proinflammatory end of the spectrum. Age‐associated increases in plasma GluCer were also positively associated with activation of the AGE‐RAGE signaling pathway. Advanced glycation end products (AGEs) denote a diverse array of compounds produced by nonenzymatic interactions between reducing sugars and associated biomolecules that include proteins, lipids, or amino acids [58].

We next created in vitro and in vivo models of GluCer accumulation using the GCase inhibitor CBE to investigate its effects on kidney metabolism. GluCer overload was previously shown to distort mitochondrial cristae morphology and interfere with mitochondrial respiration in dorsal root ganglion neurons [59]. *GBA* mutations also impair mitophagy via disrupting mitochondrial priming and autophagy induction [60]. Mitochondrial dysfunction due to prolonged M–L contacts is partially rescued by TBC1D15 expression in PD patient‐derived mutant GBA1 dopaminergic neurons [46]. Consistent with and extending these reports, we observed altered mitochondrial morphology, afflicted mitochondrial respiration, and decreased TBC1D15 expression in PCTCs isolated from old mice. Critically, using super‐resolution live‐cell imaging, we provide direct evidence that GluCer accumulation significantly prolongs M–L contact duration and disrupts interorganellar untethering dynamics in aged PCTCs, offering a direct link between GluCer overload and impaired mitochondrial quality control. Defective mitochondrial function was reversed by abrogating GluCer accumulation via the addition of EG. Mitochondria from PCTCs of young mice were resilient to GluCer overload. Of interest, we observed that the metabolomic profiles of PCTCs were reversed upon restoration of mitochondrial function by modulating GluCer bioavailability.

To elucidate resiliency mechanisms that help females cope with GluCer overload before overt renal functional decline at old age, we challenged late middle‐aged female mice with CBE. ORA analysis of enriched pathways suggests that the kidneys handle GluCer overload by increasing SLC‐mediated transmembrane transport, which translates to higher energy demand likely offset by enhanced activity of the TCA cycle and respiratory electron transport chain. An unaltered level of Pink1, however, showed that the overall mitochondrial pool likely remains functionally intact with normal membrane potential despite the activation of mTORC1. Inhibition of mTORC1 signaling regulates Pink1/Parkin‐mediated targeting of depolarized mitochondria to the autophagic machinery, and mTORC1 hyperactivation impedes mitophagy [47, 61]. The metabolic scenario in late middle‐aged female mice likely reflects early compensatory responses to renal GluCer overload. In response to ectopic GluCer accumulation, the kidneys increase purine catabolism, leading to increases in xanthine and uric acid while expending adenylates and guanylates. Both adenylates and guanylates promote mTORC1 activation [49, 62], while uric acid might have a dual effect [63, 64]. Uric acid overload reinforces mTORC1 inhibition via activating AMP‐activated protein kinase (AMPK) in PCTCs [63], but activates mTORC1 in monocytes by promoting the phosphorylation of proline‐rich AKT substrate 40 (PRAS40) [63]. The cumulative results indicate that augmented purine catabolism in PCTCs at late middle‐age may be an adaptive response to maintain adenylates and guanylates at low levels to attenuate mTORC1 activation in order to maintain mitochondrial function.

Our findings showed that the adverse effect of age‐associated GluCer accumulation on renal mitochondrial metabolism during female aging is mediated via mTOR and modulated by intracellular purine levels. Pharmacological intervention to deplete endogenous purines using clinically relevant inhibitors (MA, MTX, and 6‐MP) effectively reversed the CBE‐induced transcriptional changes in mitophagy genes and fully restored mitochondrial metabolic function, as evidenced by the rescue of basal respiration and ATP production in aged PCTCs. Our findings corroborate the preceding report that depletion of purine nucleotides suppresses mTORC1 signaling, which can be re‐stimulated upon the addition of exogenous purines [49]. Finally, we showed that the artificial induction of GluCer accumulation in male mice via CBE administration similarly triggered mTOR activation, impaired mitophagy, renal fibrosis, and significant mortality that were likewise rescued by purine depletion. Our findings demonstrate that the core GluCer‐purine‐mTORC1‐mitophagy cascade is conserved in both sexes, and that female‐specific vulnerability to age‐related renal decline is ascribed to sexually dimorphic upstream handling of GluCer, leading to preferential accumulation of GluCer in aging females. mTOR can sense changes in GluCer levels that subsequently trigger its activation [65], and GluCer‐mTOR signaling induces redistribution of peroxisomes within mammalian cells [66]. Inhibition of mTORC1 enhances longevity [67]. mTORC1 signaling also regulates mitochondrial oxidative function [67, 68, 69]. mTORC1 inhibition is required for mitophagy to ensue, and hyperactivation of mTORC1 resulting from tuberous sclerosis complex (TSC) ablation increases the occurrence of dysfunctional mitochondria loaded with oxidized mitochondrial proteins [70].

Our discovery of purine‐modulated, mTORC1‐mediated mitophagy as a conserved pathway of renal senescence, which is disproportionately activated in females due to higher GluCer overload with aging, is in agreement with the known roles of mTORC1 hyperactivity in numerous late‐onset or age‐related pathologies. For example, enhanced mTORC1 activity was shown to promote aging of the liver via triggering age‐associated defects in ketogenesis [71]. Corroborating our findings, sexual dimorphism in the effect of mTOR signaling on lifespan extension was previously reported. Female mice treated with rapamycin displayed an 18% increase in lifespan, whereas only a 10% increase was observed in males [72]. Additionally, knockout of the mTORC1‐dependent S6K1 extends lifespan specifically in female mice, with no longevity benefit for males [73]. Female‐specific renal accumulation of GluCer commencing from late middle‐age, therefore, confers a molecular explanation for the female‐biased benefits on lifespan extension with mTOR inhibition.

Furthermore, we discovered purine catabolism as a resiliency mechanism that antagonizes GluCer‐mTOR signaling in female kidneys at late middle‐age. Investigating compensatory mechanisms that attenuate aging at late middle‐age is meaningful in terms of uncovering new molecular targets for intervention. In our study, the greatest number of age‐associated metabolites occurred in the 56‐year‐old window, in agreement with preceding work on aging brain that identified the window of 50–55 years as a predominant period of metabolic transition [74]. The antagonistic pleiotropy theory of aging argues that traits conferring early reproductive advantage might elicit deleterious effects later in life [75]. The female kidneys evoke a higher metabolic cost in maintaining homeostasis of electrolytes and ions than male kidneys, ascribed to a downward shift in sodium chloride reabsorption at later segments past the proximal tubules with a heavier reliance on Na^+^K^+^‐ATPase [76]. Sex‐dependent differences in the expressions of organic solute transporters (OATs) also contribute to the enhanced malleability of female kidneys to cope with varying workload, enabling the female kidneys to adapt to changing nutritional requirements of serial pregnancies (e.g., in diverting fluids and electrolytes to the developing fetus or the mammary glands) to optimize reproduction [77]. OAT2, for example, exhibits female‐biased expression that is stimulated by estradiol and progesterone, but inhibited by testosterone [78]. The plasticity in renal function in response to changing estrogen levels during the female reproductive phase might render females liable to an abrupt decline in kidney function induced by GluCer‐mTOR signaling postreproduction when estrogen levels fall. The higher energy expenditure incurred in maintaining kidney function for females also increases female susceptibility to renal mitochondrial dysfunction. Failure to sustain such high energy demand jeopardizes renal homeostasis and initiates a downward metabolic spiral that accelerates age‐associated functional decline. Our current observations thus corroborate epidemiological findings reporting a higher prevalence of CKD in women compared with men, particularly postmenopause [79]. The lifetime risk of kidney failure, however, is higher in men than in women, which might be attributed to renoprotective resiliency mechanisms fueled by female hormones earlier in life [80].

This study has several limitations that are critical to framing key directions for future research. First, the cross‐sectional design of our human plasma cohorts precludes causal inference. It also cannot resolve whether GluCer accumulation precedes renal functional decline or arises as a downstream consequence. Longitudinal, within‐individual profiling of GluCer and uremic toxins, together with renal functional indices such as eGFR, is required to establish temporal sequence and strengthen causal interpretation. Second, our murine studies and primary PCTCs experiments enable mechanistic dissection, but they cannot fully model the complexity of human aging. These include menopause, chronic comorbidities, and lifelong environmental exposures. In addition, acute pharmacologic GCase inhibition produces rapid GluCer accumulation, which may not fully mirror the slow, multifactorial glycosphingolipid remodeling characteristic of natural aging in humans. Future work should evaluate whether gradual, chronic glycosphingolipid remodeling produces the same renal mitochondrial vulnerabilities using more physiologically relevant models (kidney tubule–specific genetic modulation of GCase/UGCG). Third, while purine depletion with clinical agents (MA, MTX, 6‐MP) to counteract senescence provides compelling proof‐of‐concept, systemic inhibition of purine metabolism and mTORC1 signaling may potentially trigger immunologic and metabolic liabilities. Future work must therefore evaluate off‐target effects, tissue‐specific responses, and long‐term safety in chronic, aged, and comorbidity‐relevant models, while also exploring optimized dosing regimens and kidney‐targeted delivery options. Addressing these limitations through longitudinal human studies and refined preclinical models will be essential for translating the purine–mTORC1 axis into safe, effective senotherapeutic interventions.

## 4 Conclusion

**FIGURE 9:**
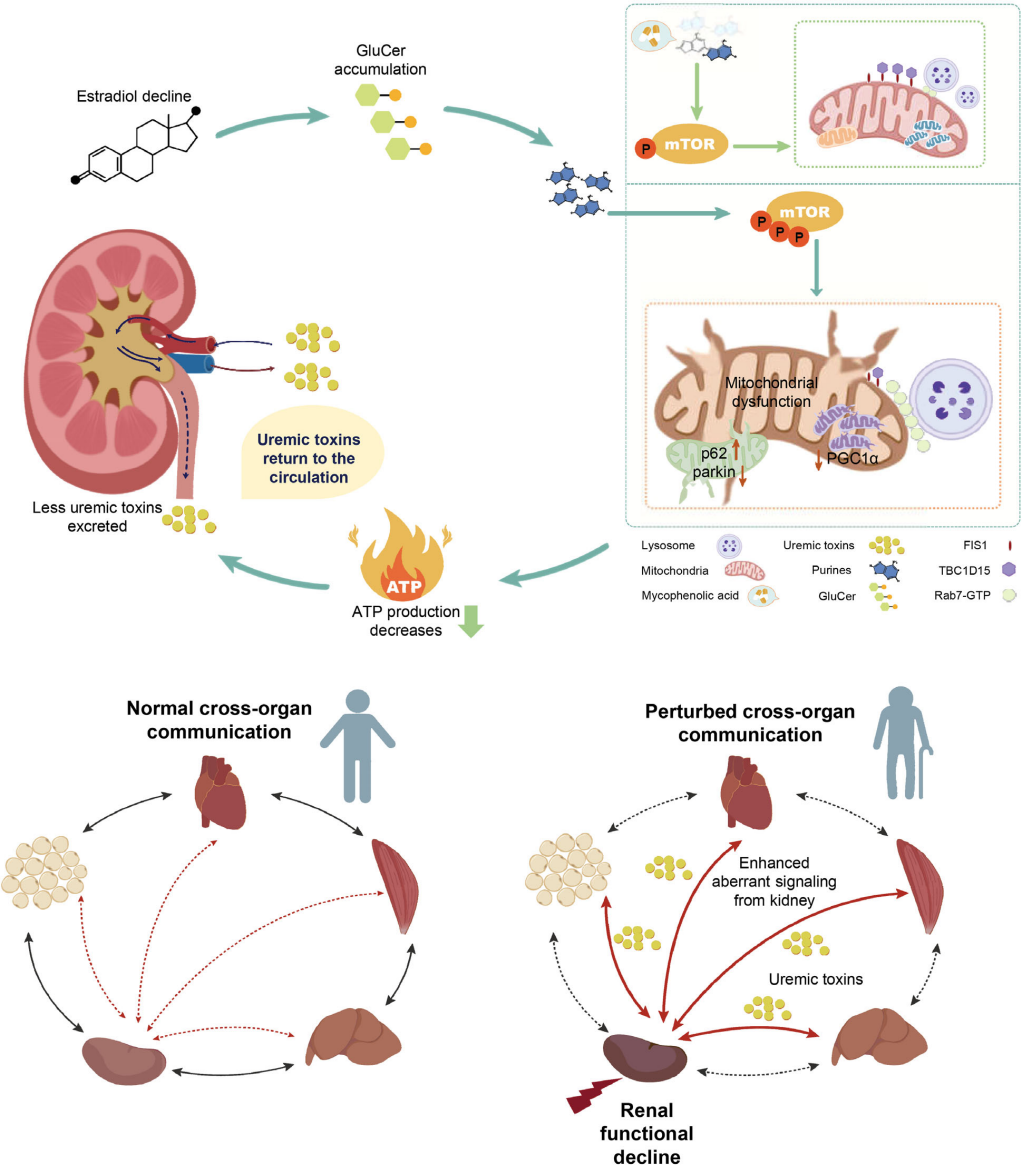
**Female‐specific renal GluCer accumulation disrupts mitochondrial quality control via a conserved purine‐mTORC1 pathway, triggering a wave of uremic toxins into the systemic circulation that constitutes a female‐biased vulnerability toward renal‐driven multiorgan senescence**. We propose that, as estrogen levels fall in late middle‐age, the inhibitory effect on GluCer synthase may be relieved, and GluCer overload commences in the female kidneys. GluCer accumulation, in concert with purine accretion ascribed to senescence‐associated reduction in purine catabolism, promotes mTOR phosphorylation and undermines mitochondrial quality control. Specifically, mTOR activation alters the expression of key mitophagy regulators—increasing p62 while decreasing parkin—and suppresses the mitochondrial biogenesis factor PGC1α, collectively contributing to mitochondrial dysfunction. Concurrently, GluCer accumulation impairs M–L untethering, evidenced by reduced TBC1D15 and Fis1 protein levels. These defects further compromise mitochondrial function and energy production, as reflected by decreased ATP generation. The ensuing renal mitochondrial failure compromises normal kidney physiology, leading to reduced urine excretion and increased reflux of uremic toxins back into the systemic circulation, thereby accelerating multiorgan senescence. Notably, administration of mycophenolic acid—an inhibitor of purine synthesis—attenuates mTOR phosphorylation and helps restore mitochondrial homeostasis, highlighting inhibitors of purine production as potential senotherapeutics for mitigating kidney‐driven systemic senescence triggered by GluCer accumulation.

Our trans‐omics approach highlights the kidney as a central node strongly linked to systemic aging (Figure 9). We demonstrate that age‐associated renal GluCer accumulation disrupts mitochondrial quality control via a conserved purine‐mTORC1 pathway, triggering a wave of uremic toxins in the systemic circulation that accelerates multiorgan senescence. This vulnerability is amplified in aging females due to higher GluCer load, potentially driven by the sharp postmenopausal decline in estrogen levels that dysregulate glycosphingolipid‐metabolizing enzymes [54], which may denote an evolutionary trade‐off between somatic maintenance and successful reproduction earlier in life. Critically, we showed that the GluCer‐triggered defects in mitophagy and mitochondrial dysfunction can be rescued by pharmacological depletion of purines using clinically relevant inhibitors. Our findings position existing purine‐modulating drugs as promising senotherapeutic candidates to mitigate renal decline, thereby slowing down a key driver of systemic aging.

## 5 Methods

### 5.1 Study Design and Participants

#### 5.1.1 Cross‐Sectional Cohort

This study was approved by the Ethics Committee of Beijing Anzhen Hospital, Capital Medical University, approval number 2018010. Demographics of the participants were presented in Table 1.

#### 5.1.2 Longitudinal Cohort

Both the clinical trial and the extended follow‐up study were approved by the medical ethics committee of the Chinese University of Hong Kong and the New Territories East Cluster of Hospital Authority of Hong Kong, and the trial was registered at the Clinical Trial Registry of the US (NCT02457507). Demographics of the study participants were presented in Table 2.

### 5.2 Polar Metabolite Extraction

Polar metabolites were extracted from plasma as previously described [16, 17]. Briefly, 50 µL of plasma were mixed with 200 µL of ice‐cold methanol containing 0.37 mM phenylhydrazine, vortexed for 10 s, and incubated for 30 min at 1500 rpm and 4°C in an orbital shaker, then centrifuged for 10 min at 12,000 rpm and 4°C. The supernatant was transferred into a clean 1.5 mL centrifuge tube and dried using a SpeedVac (Genevac miVac, Tegent Scientific Ltd., England). The dried extracts were redissolved with 2% acetonitrile in water, centrifuged for 2 min at 9000 rpm, and the clean supernatant was collected for LC‐MS analysis.

### 5.3 Lipid Extraction

Lipids were extracted using a modified version of Bligh and Dyer's protocol [17]. Ice‐cold chloroform:methanol (1:2, v/v), 750 µL, was added to 100 µL plasma in a fresh 1.5 mL Eppendorf brand safe‐lock tube placed on ice. The samples were vortexed for 15 s and incubated for 30 min in a 4°C cold room at 1500 rpm. At the end of incubation, 350 µL of ice‐cold deionized H_2_O and 250 µL of ice‐cold chloroform were added to induce phase separation. The lower organic phase was transferred to a fresh tube after centrifugation at 12,000 rpm for 5 min at 4°C. A second round of extraction was performed via the addition of 450 µL of ice‐cold chloroform to the remaining samples. The samples were vortexed and centrifuged at 12,000 rpm for 5 min at 4°C. The organic extracts from both rounds of extraction were pooled and dried using SpeedVac (Genevac miVac, Tegent Scientific Ltd.) under OH mode for lipidomic analyses.

### 5.4 Animals

C57BL/6 N male and female mice were purchased from Beijing Vital River Laboratory Animal Technologies Co., Ltd. A 12 h light–dark cycle was maintained in the housing room. The temperature was set as 22 ± 1°C. All mice had access to food and water ad libitum, and each mouse was housed in a separate cage. All mice were fed with a standard AIN‐93M diet.

### 5.5 Lipid and Metabolite Extraction from Mouse Tissues

Lipids and metabolites were extracted from tissues using established protocols [81, 82]. Detailed descriptions are provided in the Methods section of the Supporting Information.

### 5.6 Metabolomics

Positive and negative polarity data were acquired on the Agilent 6546 LC/Q‐TOF using full scan mode with ranges of m/z 60–1100. Data‐dependent acquisition using iterative exclusion was conducted via five repeated injections of a pooled (QC) plasma sample for each polarity [83]. Peak areas of endogenous metabolites were normalized to the areas of their corresponding isotopically‐labeled structural analogues for quantitation [16, 17]. Metabolites were annotated and reported using three confidence levels following commonly used community recommendations [84, 85]. Level 1 indicates metabolites confirmed by matching MS1 accurate mass, retention time, and MS/MS spectra to authentic standards analyzed under the same conditions. Level 2 indicates metabolites putatively annotated by matching MS1 and MS/MS spectra to public metabolite spectral libraries without RT confirmation by in‐house standards. Level 3 indicates metabolites annotated based on MS1 and MS/MS spectral similarity to known compounds within a chemical class, or based on MS1 features combined with database searches when MS/MS evidence was insufficient. A total of 229 metabolites were annotated from mouse kidney tissues, with 127 metabolites at confidence level 1 (56 %), 92 metabolites at confidence level 2 (40 %), and 10 metabolites at confidence level 3 (4 %) (Table S20). Detailed descriptions are provided in the Methods section of the Supporting Information.

### 5.7 Lipidomics

Analyses of polar lipids from murine tissue and organ lipid extracts were performed on a Jasper HPLC coupled with Sciex Triple Quad 4500MD, whereas neutral lipids were analyzed on an Agilent 1260 HPLC connected to Sciex 5500 QTRAP, both under electrospray ionization mode. Methodological details were comprehensively reported in a recent publication [86]. Detailed descriptions are provided in the Methods section of the Supporting Information.

### 5.8 Immunoblot Analysis

Kidneys were dissected and snap‐frozen in liquid nitrogen. For cultured cells, the cells were centrifuged, the medium was removed, and the pellet was snap‐frozen in liquid nitrogen. Proteins from tissues and cell pellets were extracted using RIPA lysis buffer (Meilunbio) with protease and phosphatase inhibitor cocktail (Meilunbio). The protein content was determined using a Pierce BCA protein assay kit (Thermo Fisher Scientific) according to the manufacturer's instructions. After boiling, lysates containing 40 µg protein were subjected to SDS‐PAGE and transblotted onto nitrocellulose membranes (Pall). Primary antibodies included: anti‐p53 (Cell Signaling, 2524S, 1:500), anti‐OPA1 (abcam, ab42364, 1:1000), anti‐DRP1 (Cell Signaling, 8570, 1:1000), anti‐mTOR (Immunoway, YT2913, 1:1000), anti‐p‐mTOR (Abmart, T56571, 1:1000), anti‐S6K (Cell Signaling, 9202S, 1:1000), anti‐p‐S6K (Cell Signaling, 9205S, 1:1000), anti‐β‐actin (Sigma, A5441, 1:1000), anti‐TBC1D15 (Immunoway, YN3051, 1:1000), anti‐pink1 (Santa Cruz, sc‐517353,1:50), anti‐α‐Tubulin (Proteintech, 66031‐1‐Ig, 1:1000), anti‐FIS1 (Proteintech,10956‐1‐AP, 1:1000), anti‐p62 (Immunoway, YM8025, 1:1000), anti‐LC3 (Proteintech, 14600‐1‐AP, 1:1000), anti‐Parkin (Proteintech, 14060‐1‐AP, 1:1000), and anti‐GAPDH (CUSABIO, CSB‐MA000071M1m, 1:2000). Secondary antibodies included goat anti‐rabbit HRP (ZSGB‐BIO, ZB‐2305, 1:5000) and goat anti‐mouse HRP (ZSGB‐BIO, ZB‐2301, 1:5000). Chemiluminescence detection of proteins was performed with super sensitive ECL luminescence reagent (Meilunbio) and imaged with the Chemidoc Imaging System (Bio‐Rad). Bands were quantified using ImageJ software.

### 5.9 Quantitative RT‐PCR

Total RNA was isolated using TRIzol reagents (Cat#15,596,018, Thermo Fisher Scientific) according to the manufacturer's instructions (Invitrogen, USA). cDNA was generated from 0.5µg RNA as a template using a reverse transcription kit (MJ Mini, Bio‐Rad). Reverse transcription products were quantified by real‐time PCR (LightCycler 480, Roche) in a final reaction volume of 20µl using SYBR Green. The sequence of primers used to amplify the cDNA is reported in Table S19.

### 5.10 Bioinformatics and Statistical Analyses

Metabolites peak areas were log‐transformed and standardized to z‐scores. Detailed descriptions are provided in the Methods section of the Supporting Information. DE‐SWAN was used to detect nonlinear, stage‐specific age effects [33]. Instead of fitting a single global age trend, DE‐SWAN iteratively centers a sliding age window (e.g., 20 years) at each age and compares molecular levels between two adjacent age parcels within that window (e.g., [age−10, age] vs. [age, age+10]) using a regression model with relevant covariates. By repeating this comparison while sliding the window across the lifespan (typically in 1‐year increments), DE‐SWAN quantifies local differential changes at each age, enabling the identification of discrete “waves/crests” where many features shift simultaneously—an approach particularly suitable for capturing nonlinear aging trajectories that can be obscured by global linear modeling. Clusters of co‐abundant plasma metabolites and lipids were identified using the R package WGCNA as described previously [87, 88]. All the statistical analyses were performed using R software version 4.3.2.

## Author Contributions

G.S. and S.M.L. conceived, designed, and supervised experiments. Z.N., C.C., Y.T., H.T., Z.W., S.Z., M.C., J.M., H.S., H.C., and H. M. performed the experiments. J. C., Y.Y., and Z.N. performed the statistical analysis. G.S., S.M.L., Y.W., and J.S. interpreted data. Z.N., S.M.L., W.L.F.L., and G.S. wrote and edited the manuscript. Y.W., J.D., and T.K. recruited clinical samples. All authors read and approved the final manuscript.

## Funding

This work was financially supported by grants from the National Natural Science Foundation of China to G.S. (32321004 and 92357308), the Chinese Academy of Sciences (XDB39050900) to G.S., and the National Key R&D Program of China to S.M.L. (2022YFA0806001).

## Ethics Statement

All animal handling procedures were approved by the Animal Care and Use Committee of the Institute of Genetics and Developmental Biology, Chinese Academy of Sciences (approval no. AP2022012).

## Conflicts of Interest

Wei Ling Florence Lim, Jingkang Cui, and Sin Man Lan are employees of LipidALL Technologies. The remaining authors declare no conflicts of interest.

## Supporting information



Supporting information

Supporting information

## Data Availability

All source datasets were provided as Supplementary tables. All other data will be provided by the corresponding author upon reasonable request.

## References

[1] T. Niccoli and L. Partridge , “Ageing as a Risk Factor for Disease”. Current Biology 22, (2012): R741–R752. 22975005 10.1016/j.cub.2012.07.024

[2] Y. E. Tian , V. Cropley , A. B. Maier , N. T. Lautenschlager , M. Breakspear , and A. Zalesky , “Heterogeneous Aging Across Multiple Organ Systems and Prediction of Chronic Disease and Mortality,” Nature Medicine 29, (2023): 1221–1231. 10.1038/s41591-023-02296-6 37024597

[3] T. Harayama and H. Riezman , “Understanding the Diversity of Membrane Lipid Composition,” Nature Reviews Molecular Cell Biology 19, (2018): 281–296. 29410529 10.1038/nrm.2017.138

[4] R. Laaksonen , K. Ekroos , M. Sysi‐Aho , et al., “Plasma Ceramides Predict Cardiovascular Death in Patients with Stable Coronary Artery Disease and Acute Coronary Syndromes Beyond LDL‐Cholesterol,” European Heart Journal 37, (2016): 1967–1976. 27125947 10.1093/eurheartj/ehw148 PMC4929378

[5] A. Mantovani , S. Bonapace , G. Lunardi , et al., “Associations Between Specific Plasma Ceramides and Severity of Coronary‐Artery Stenosis Assessed by Coronary Angiography,” Diabetes & Metabolism 46, (2020): 150–157. 31386900 10.1016/j.diabet.2019.07.006

[6] A. M. Fretts , P. N. Jensen , A. N. Hoofnagle , et al., “Plasma Ceramides Containing Saturated Fatty Acids Are Associated with Risk of Type 2 Diabetes,” Journal of Lipid Research 62, (2021): 100119. 34555371 10.1016/j.jlr.2021.100119 PMC8517199

[7] P. J. Meikle , G. Wong , C. K. Barlow , et al., “Plasma Lipid Profiling Shows Similar Associations with Prediabetes and Type 2 Diabetes,” PLoS ONE 8, (2013): e74341. 24086336 10.1371/journal.pone.0074341 PMC3785490

[8] K. Huynh , W. L. F. Lim , C. Giles , et al., “Concordant Peripheral Lipidome Signatures in Two Large Clinical Studies of Alzheimer's Disease,” Nature Communications 11, (2020): 5698. 10.1038/s41467-020-19473-7 PMC7655942 33173055

[9] X. Han , D. Holtzman , and D. McKeel Jr. , “Plasmalogen Deficiency in Early Alzheimer's Disease Subjects and in Animal Models: Molecular Characterization Using Electrospray Ionization Mass Spectrometry,” Journal of Neurochemistry 77, (2001): 1168–1180. 11359882 10.1046/j.1471-4159.2001.00332.x

[10] R. G. Cutler , J. Kelly , K. Storie , et al., “Involvement of Oxidative Stress‐Induced Abnormalities in Ceramide and Cholesterol Metabolism in Brain Aging and Alzheimer's Disease,” Proceedings of the National Academy of Sciences 101, (2004): 2070–2075. 10.1073/pnas.0305799101 PMC357053 14970312

[11] A. Michalczyk , B. Dołęgowska , R. Heryć , D. Chlubek , and K. Safranow , “Associations Between Plasma Lysophospholipids Concentrations, Chronic Kidney Disease and the Type of Renal Replacement Therapy,” Lipids Health Diseases 18, (2019): 85. 10.1186/s12944-019-1040-5 PMC6449907 30947711

[12] Y. Yamaguchi , M. Zampino , R. Moaddel , et al., “Plasma Metabolites Associated with Chronic Kidney Disease and Renal Function in Adults from the Baltimore Longitudinal Study of Aging,” Metabolomics 17, (2021): 9. 33428023 10.1007/s11306-020-01762-3 PMC9220986

[13] K. A. Lawton , A. Berger , M. Mitchell , et al., “Analysis of the Adult Human Plasma Metabolome,” Pharmacogenomics 9, (2008): 383–397. 18384253 10.2217/14622416.9.4.383

[14] S. Ahadi , W. Zhou , S. M. Schüssler‐Fiorenza Rose , et al., “Personal Aging Markers and Ageotypes Revealed by Deep Longitudinal Profiling,” Nature Medicine 26, (2020): 83–90. 10.1038/s41591-019-0719-5 PMC7301912 31932806

[15] R. Wang , B. Li , S. Lam , and G. Shui , “Integration of Lipidomics and Metabolomics for In‐Depth Understanding of Cellular Mechanism and Disease Progression,” Journal of Genetics and Genomics 47, (2020): 69–83. 32178981 10.1016/j.jgg.2019.11.009

[16] H. Tian , Z. Ni , S. M. Lam , et al., “Precise Metabolomics Reveals a Diversity of Aging‐Associated Metabolic Features,” Small Methods 6, (2022): e2200130. 35527334 10.1002/smtd.202200130

[17] S. M. Lam , C. Zhang , Z. Wang , et al., “A Multi‐Omics Investigation of the Composition and Function of Extracellular Vesicles Along the Temporal Trajectory of COVID‐19,” Nature Metabolism 3, (2021): 909–922. 10.1038/s42255-021-00425-4 34158670

[18] O. Goek , A. Döring , C. Gieger , et al., “Serum Metabolite Concentrations and Decreased GFR in the General Population,” American Journal of Kidney Diseases 60, (2012): 197–206. 22464876 10.1053/j.ajkd.2012.01.014

[19] W. Guder and S. Wagner , “The Role of the Kidney in Carnitine Metabolism,” Journal of Clinical Chemistry and Clinical Biochemistry 28, (1990): 347–350. 2199595

[20] B. Hocher and J. Adamski , “Metabolomics for Clinical Use and Research in Chronic Kidney Disease,” Nature Reviews Nephrology 13, (2017): 269–284. 28262773 10.1038/nrneph.2017.30

[21] C. McCoin , T. Knotts , and S. Adams , “Acylcarnitines–Old Actors Auditioning for New Roles in Metabolic Physiology,” Nature Reviews Endocrinology 11, (2015): 617–625. 10.1038/nrendo.2015.129 PMC4966159 26303601

[22] N. Fujiwara , H. Nakagawa , K. Enooku , et al., “CPT2 Downregulation Adapts HCC to Lipid‐Rich Environment and Promotes Carcinogenesis via Acylcarnitine Accumulation in Obesity,” Gut 67, (2018): 1493–1504. 29437870 10.1136/gutjnl-2017-315193 PMC6039238

[23] X. Wang , S. Yang , S. Li , et al., “Aberrant Gut Microbiota Alters Host Metabolome and Impacts Renal Failure in Humans and Rodents,” Gut 69, (2020): 2131–2142. 32241904 10.1136/gutjnl-2019-319766 PMC7677483

[24] K. Saito , S. Fujigaki , M. P. Heyes , et al., “Mechanism of Increases in L‐Kynurenine and Quinolinic Acid in Renal Insufficiency,” American Journal of Physiology Renal Physiology 279, (2000): F565–F572. 10966936 10.1152/ajprenal.2000.279.3.F565

[25] W. Hassan , P. Shrestha , K. Sumida , et al., “Association of Uric Acid‐Lowering Therapy With Incident Chronic Kidney Disease,” JAMA Network Open 5, (2022): e2215878. 35657621 10.1001/jamanetworkopen.2022.15878 PMC9166229

[26] R. Vanholder , R. De Smet , G. Glorieux , et al., “Review on Uremic Toxins: Classification, Concentration, and Interindividual Variability,” Kidney International 63, (2003): 1934–1943. 12675874 10.1046/j.1523-1755.2003.00924.x

[27] S. Luo , A. Surapaneni , Z. Zheng , et al., “NAT8 Variants, N‐Acetylated Amino Acids, and Progression of CKD,” Clinical Journal of American Society of Nephrology 16, (2020): 37–47. 10.2215/CJN.08600520 PMC7792648 33380473

[28] S. Luo , E. V. Feofanova , A. Tin , et al., “Genome‐wide Association Study of Serum Metabolites in the African American Study of Kidney Disease and Hypertension,” Kidney International 100, (2021): 430–439. 33838163 10.1016/j.kint.2021.03.026 PMC8583323

[29] J. C. Chambers , W. Zhang , G. M. Lord , et al., “Genetic Loci Influencing Kidney Function and Chronic Kidney Disease,” Nature Genetics 42, (2010): 373–375. 20383145 10.1038/ng.566 PMC3748585

[30] K. Yoshioka , Y. Hirakawa , M. Kurano , et al., “Lysophosphatidylcholine Mediates Fast Decline in Kidney Function in Diabetic Kidney Disease,” Kidney International 101, (2022): 510–526. 34856312 10.1016/j.kint.2021.10.039

[31] D. Wen , Z. Zheng , A. Surapaneni , et al., “Metabolite Profiling of CKD Progression in the Chronic Renal Insufficiency Cohort Study,” JCI Insight 7, (2022): e161696. 36048534 10.1172/jci.insight.161696 PMC9714776

[32] H. Lindner , M. Täfler‐Naumann , and K. Röhm , “N‐Acetylamino Acid Utilization by Kidney Aminoacylase‐1,” Biochimie 90, (2008): 773–780. 18222180 10.1016/j.biochi.2007.12.006

[33] B. Lehallier , D. Gate , N. Schaum , et al., “Undulating Changes in Human Plasma Proteome Profiles Across the Lifespan,” Nature Medicine 25, (2019): 1843–1850. 10.1038/s41591-019-0673-2 PMC7062043 31806903

[34] S. J. Mitchell , M. Bernier , M. A. Aon , et al., “Nicotinamide Improves Aspects of Healthspan, but Not Lifespan, in Mice,” Cell Metabolism 27, (2018): 667–676. 29514072 10.1016/j.cmet.2018.02.001 PMC5854409

[35] D. Shan , Y. Wang , Y. Chang , et al., “Dynamic Cellular Changes in Acute Kidney Injury Caused by Different Ischemia Time,” iScience 26, (2023): 106646. 37168554 10.1016/j.isci.2023.106646 PMC10165188

[36] H. Wang , A. Ainiwaer , Y. Song , et al., “Perturbed Gut Microbiome and Fecal and Serum Metabolomes Are Associated With Chronic Kidney Disease Severity,” Microbiome 11, (2023): 3. 36624472 10.1186/s40168-022-01443-4 PMC9827681

[37] F. Guebre‐Egziabher , P. M. Alix , L. Koppe , et al., “Ectopic Lipid Accumulation: A Potential Cause for Metabolic Disturbances and a Contributor to the Alteration of Kidney Function,” Biochimie 95, (2013): 1971–1979. 23896376 10.1016/j.biochi.2013.07.017

[38] M. Brosnan and J. Brosnan , “Renal Arginine Metabolism,” Journal of Nutrition 134, (2004): 2791s–2795s. 15465786 10.1093/jn/134.10.2791S

[39] M. M. Rinschen , O. Palygin , A. El‐Meanawy , et al., “Accelerated Lysine Metabolism Conveys Kidney Protection in Salt‐Sensitive Hypertension,” Nature Communications 13, (2022): 4099. 10.1038/s41467-022-31670-0 PMC9283537 35835746

[40] S. Lam , R. Wang , H. Miao , B. Li , and G. Shui , “An Integrated Method for Direct Interrogation of Sphingolipid Homeostasis in the Heart and Brain Tissues of Mice Through Postnatal Development up to Reproductive Senescence,” Analytica Chimica Acta 1037, (2018): 152–158. 30292289 10.1016/j.aca.2018.01.015

[41] D. Dominissini , S. Nachtergaele , S. Moshitch‐Moshkovitz , et al., “The Dynamic N(1)‐Methyladenosine Methylome in Eukaryotic Messenger RNA,” Nature 530, (2016): 441–446. 26863196 10.1038/nature16998 PMC4842015

[42] E. Mishima , C. Inoue , D. Saigusa , et al., “Conformational Change in Transfer RNA Is an Early Indicator of Acute Cellular Damage,” Journal of the American Society of Nephrology 25, (2014): 2316–2326. 24833129 10.1681/ASN.2013091001 PMC4178440

[43] N. Schömel , G. Geisslinger , and M. Wegner , “Influence of Glycosphingolipids on Cancer Cell Energy Metabolism,” Progress in Lipid Research 79, (2020): 101050. 32592726 10.1016/j.plipres.2020.101050

[44] C. Kuo , W. W. Kallemeijn , L. T. Lelieveld , et al., “In vivo inactivation of Glycosidases by Conduritol B Epoxide and Cyclophellitol as Revealed by Activity‐Based Protein Profiling,” FEBS Journal 286, (2019): 584–600. 30600575 10.1111/febs.14744 PMC6850446

[45] S. Chen , R. Liu , C. Mo , et al., “Multi‐Omic and Spatial Analysis of Mouse Kidneys Highlights Sex‐Specific Differences in Gene Regulation Across the Lifespan,” Nature Genetics 57, (2025): 1213–1227. 40259083 10.1038/s41588-025-02161-x PMC12081296

[46] S. Kim , Y. Wong , F. Gao , and D. Krainc , “Dysregulation of Mitochondria‐Lysosome Contacts by GBA1 Dysfunction in Dopaminergic Neuronal Models of Parkinson's Disease,” Nature Communications 12, (2021): 1807. 10.1038/s41467-021-22113-3 PMC7985376 33753743

[47] A. Bartolomé , A. García‐Aguilar , S. I. Asahara , et al., “MTORC1 Regulates Both General Autophagy and Mitophagy Induction After Oxidative Phosphorylation Uncoupling,” Molecular and Cellular Biology 37, (2017): e00441–17. 28894028 10.1128/MCB.00441-17 PMC5686580

[48] D. P. Narendra , S. M. Jin , A. Tanaka , et al., “PINK1 is Selectively Stabilized on Impaired Mitochondria to Activate Parkin,” PLoS Biology 8, (2010): e1000298. 20126261 10.1371/journal.pbio.1000298 PMC2811155

[49] G. Hoxhaj , J. Hughes‐Hallett , R. C. Timson , et al., “The mTORC1 Signaling Network Senses Changes in Cellular Purine Nucleotide Levels,” Cell Reports 21, (2017): 1331–1346. 29091770 10.1016/j.celrep.2017.10.029 PMC5689476

[50] P. Bhargava and R. Schnellmann , “Mitochondrial Energetics in the Kidney,” Nature Reviews Nephrology 13, (2017): 629–646. 28804120 10.1038/nrneph.2017.107 PMC5965678

[51] S. Nigam and K. Bush , “Uraemic Syndrome of Chronic Kidney Disease: Altered Remote Sensing and Signalling,” Nature Reviews Nephrology 15, (2019): 301–316. 30728454 10.1038/s41581-019-0111-1 PMC6619437

[52] R. T. Gansevoort , R. Correa‐Rotter , B. R. Hemmelgarn , et al., “Chronic Kidney Disease and Cardiovascular Risk: Epidemiology, Mechanisms, and Prevention,” Lancet 382, (2013): 339–352. 23727170 10.1016/S0140-6736(13)60595-4

[53] P. Stevens , D. O'Donoghue , S. de Lusignan , et al., “Chronic Kidney Disease Management in the United Kingdom: NEOERICA Project Results,” Kidney International 72, (2007): 92–99. 17440495 10.1038/sj.ki.5002273

[54] A. Shukla , G. Shukla , and N. Radin , “Control of Kidney Size by Sex‐Hormones: Possible Involvement of Glucosylceramide,” American Journal of Physiology 262, (1992): F24–F29. 1733293 10.1152/ajprenal.1992.262.1.F24

[55] T. L. Habermehl , K. B. Underwood , K. D. Welch , et al., “Aging‐associated Changes in Motor Function Are Ovarian Somatic Tissue‐Dependent, but Germ Cell and Estradiol Independent in Post‐Reproductive Female Mice Exposed to Young Ovarian Tissue,” Geroscience 44, (2022): 2157–2169. 35349034 10.1007/s11357-022-00549-9 PMC8962938

[56] J. Aerts and C. Hollak , “Plasma and Metabolic Abnormalities in Gaucher's Disease,” Bailliere's Clinical Haematology 10, (1997): 691–709. 9497858 10.1016/s0950-3536(97)80034-0

[57] R. G. Boot , M. Verhoek , M. Langeveld , et al., “CCL18: A Urinary Marker of Gaucher Cell Burden in Gaucher Patients,” Journal of Inherited Metabolic Disease 29, (2006): 564–571. 16736095 10.1007/s10545-006-0318-8

[58] M. Steenbeke , R. Speeckaert , S. Desmedt , G. Glorieux , J. R. Delanghe , and M. M. Speeckaert , “The Role of Advanced Glycation End Products and Its Soluble Receptor in Kidney Diseases,” International Journal of Molecular Sciences 23, (2022): 3439. 35408796 10.3390/ijms23073439 PMC8998875

[59] L. Valek , B. Tran , A. Wilken‐Schmitz , et al., “Prodromal Sensory Neuropathy in Pink1(‐/‐) SNCA(A53T) Double Mutant Parkinson Mice,” Neuropathology and Applied Neurobiology 47, (2021): 1060–1079. 33974284 10.1111/nan.12734

[60] H. Li , A. Ham , T. C. Ma , et al., “Mitochondrial Dysfunction and Mitophagy Defect Triggered by Heterozygous GBA Mutations,” Autophagy 15, (2019): 113–130. 30160596 10.1080/15548627.2018.1509818 PMC6287702

[61] X. Li , H. Zhang , J. Li , et al., “mTORC1‐USP30‐LEF1 Cascade Regulates Cancer Stemness and Malignant Progression Through Mitonuclear Crosstalk,” MedComm 6, (2025): e70499. 41306556 10.1002/mco2.70499 PMC12644247

[62] N. Emmanuel , S. Ragunathan , Q. Shan , et al., “Purine Nucleotide Availability Regulates mTORC1 Activity Through the Rheb GTPase,” Cell Reports 19, (2017): 2665–2680. 28658616 10.1016/j.celrep.2017.05.043

[63] T. O. Crişan , M. C. P. Cleophas , B. Novakovic , et al., “Uric Acid Priming in Human Monocytes Is Driven by the AKT‐PRAS40 Autophagy Pathway,” Proceedings of National Academy of Sciences 114, (2017): 5485–5490. 10.1073/pnas.1620910114 PMC5448210 28484006

[64] J. Xiao , S. Zhu , H. Guan , et al., “AMPK Alleviates High Uric Acid‐induced Na(+)‐K(+)‐ATPase Signaling Impairment and Cell Injury in Renal Tubules,” Experimental & Molecular Medicine 51, (2019): 1–14. 10.1038/s12276-019-0254-y PMC6531502 31118410

[65] H. Zhu , H. Shen , A. Sewell , M. Kniazeva , and M. Han , “A Novel Sphingolipid‐TORC1 Pathway Critically Promotes Postembryonic Development in *Caenorhabditis elegans* ,” Elife 2, (2013): e00429. 23705068 10.7554/eLife.00429 PMC3660743

[66] N. Li , B. Hua , Q. Chen , et al., “A Sphingolipid‐mTORC1 Nutrient‐sensing Pathway Regulates Animal Development by an Intestinal Peroxisome Relocation‐Based Gut‐Brain Crosstalk,” Cell Reports 40, (2022): 111140. 35905721 10.1016/j.celrep.2022.111140

[67] S. Johnson , P. Rabinovitch , and M. Kaeberlein , “mTOR Is a Key Modulator of Ageing and Age‐Related Disease,” Nature 493, (2013): 338–345. 23325216 10.1038/nature11861 PMC3687363

[68] J. T. Cunningham , J. T. Rodgers , D. H. Arlow , F. Vazquez , V. K. Mootha , and P. Puigserver , “mTOR Controls Mitochondrial Oxidative Function Through a YY1‐PGC‐1alpha Transcriptional Complex,” Nature 450, (2007): 736–740. 18046414 10.1038/nature06322

[69] L. Ye , X. Fu , and Q. Li , “Mitochondrial Quality Control in Health and Disease,” MedComm 6 (2025): e70319. 40821693 10.1002/mco2.70319 PMC12356995

[70] A. Bartolomé , M. Kimura‐Koyanagi , S. Asahara , et al., “Pancreatic β‐Cell Failure Mediated by mTORC1 Hyperactivity and Autophagic Impairment,” Diabetes 63, (2014): 2996–3008. 24740570 10.2337/db13-0970

[71] S. Sengupta , T. Peterson , M. Laplante , S. Oh , and D. Sabatini , “mTORC1 Controls Fasting‐Induced Ketogenesis and Its Modulation by Ageing,” Nature 468, (2010): 1100–U1502. 21179166 10.1038/nature09584

[72] R. A. Miller , D. E. Harrison , C. M. Astle , et al., “Rapamycin, but Not Resveratrol or Simvastatin, Extends Life Span of Genetically Heterogeneous Mice,” Journals of Gerontology Series A, Biological Sciences and Medical Sciences 66, (2011): 191–201. 20974732 10.1093/gerona/glq178 PMC3021372

[73] C. Selman , J. M. A. Tullet , D. Wieser , et al., “Ribosomal Protein S6 Kinase 1 Signaling Regulates Mammalian Life Span,” Science 326, (2009): 140–144. 19797661 10.1126/science.1177221 PMC4954603

[74] Q. Yu , Z. He , D. Zubkov , et al., “Lipidome Alterations in Human Prefrontal Cortex During Development, Aging, and Cognitive Disorders,” Molecular Psychiatry 25, (2020): 2952–2969. 30089790 10.1038/s41380-018-0200-8 PMC7577858

[75] T. Kirkwood and M. Rose , “Evolution of Senescence: Late Survival Sacrificed for Reproduction,” Philosophical Transactions of the Royal Society of London Series B: Biological Sciences 332, (1991): 15–24. 1677205 10.1098/rstb.1991.0028

[76] L. C. Veiras , A. C. Girardi , J. Curry , et al., “Sexual Dimorphic Pattern of Renal Transporters and Electrolyte Homeostasis,” Journal of the American Society of Nephrology 28, (2017): 3504–3517. 28774999 10.1681/ASN.2017030295 PMC5698077

[77] A. McDonough , A. Harris , L. Xiong , and A. Layton , “Sex Differences in Renal Transporters: Assessment and Functional Consequences,” Nature Reviews Nephrology (2023): 21–36. 37684523 10.1038/s41581-023-00757-2 PMC11090267

[78] M. Ljubojević , D. Balen , D. Breljak , et al., “Renal Expression of Organic Anion Transporter OAT2 in Rats and Mice Is Regulated by Sex Hormones,” American Journal of Physiology‐Renal Physiology 292, (2007): F1302–F1302. 10.1152/ajprenal.00207.2006 16885152

[79] D. Murphy , C. E. McCulloch , F. Lin , et al., “Trends in Prevalence of Chronic Kidney Disease in the United States,” Annals of Internal Medicine 165, (2016): 473–481. 27479614 10.7326/M16-0273 PMC5552458

[80] J. Carrero , M. Hecking , N. Chesnaye , and K. Jager , “Sex and Gender Disparities in the Epidemiology and Outcomes of Chronic Kidney Disease,” Nature Reviews Nephrology 14, (2018): 151–164. 29355169 10.1038/nrneph.2017.181

[81] J. Song , S. M. Lam , X. Fan , et al., “Omics‐Driven Systems Interrogation of Metabolic Dysregulation in COVID‐19 Pathogenesis,” Cell Metabolism 32, (2020): 188–202.e5. 32610096 10.1016/j.cmet.2020.06.016 PMC7311890

[82] S. M. Lam , J. Li , H. Sun , et al., “Quantitative Lipidomics and Spatial MS‐Imaging Uncovered Neurological and Systemic Lipid Metabolic Pathways Underlying Troglomorphic Adaptations in Cave‐Dwelling Fish,” Molecular Biology and Evolution 39 (2022): msac050. 35277964 10.1093/molbev/msac050 PMC9011034

[83] J. P. Koelmel , X. Li , S. M. Stow , et al., “Lipid Annotator: Towards Accurate Annotation in Non‐Targeted Liquid Chromatography High‐Resolution Tandem Mass Spectrometry (LC‐HRMS/MS) Lipidomics Using a Rapid and User‐Friendly Software,” Metabolites 10 (2020): 101. 32178227 10.3390/metabo10030101 PMC7142889

[84] A. Schrimpe‐Rutledge , S. Codreanu , S. Sherrod , and J. McLean , “Untargeted Metabolomics Strategies—Challenges and Emerging Directions,” Journal of the American Society for Mass Spectrometry 27, (2016): 1897–1905. 27624161 10.1007/s13361-016-1469-y PMC5110944

[85] L. W. Sumner , A. Amberg , D. Barrett , et al., “Proposed Minimum Reporting Standards for Chemical Analysis,” Metabolomics 3, (2007): 211–221. 24039616 10.1007/s11306-007-0082-2 PMC3772505

[86] H. Miao , B. Li , Z. Wang , et al., “Lipidome Atlas of the Developing Heart Uncovers Dynamic Membrane Lipid Attributes Underlying Cardiac Structural and Metabolic Maturation,” Research 2022 (2022): 0006. 39290970 10.34133/research.0006 PMC11407523

[87] P. Langfelder and S. Horvath , “WGCNA: An R Package for Weighted Correlation Network Analysis,” BMC Bioinformatics 9, (2008): 559. 19114008 10.1186/1471-2105-9-559 PMC2631488

[88] H. K. Pedersen , V. Gudmundsdottir , H. B. Nielsen , et al., “Human Gut Microbes Impact Host Serum Metabolome and Insulin Sensitivity,” Nature 535, (2016): 376–381. 27409811 10.1038/nature18646

