# Supplementary material for: Trans‐Omics Integration Reveals That the Kidney Contributes to Systemic Aging via Sexually Dimorphic Accumulation of Glycosphingolipids
Source: MedComm (2020). 2026 Mar 7;7(3):e70669. doi: 10.1002/mco2.70669 (PMC12966803; doi:10.1002/mco2.70669)
Supplement: Supplementary file 2 — Supporting information [file MCO2-7-e70669-s002.pdf]

Figure S1

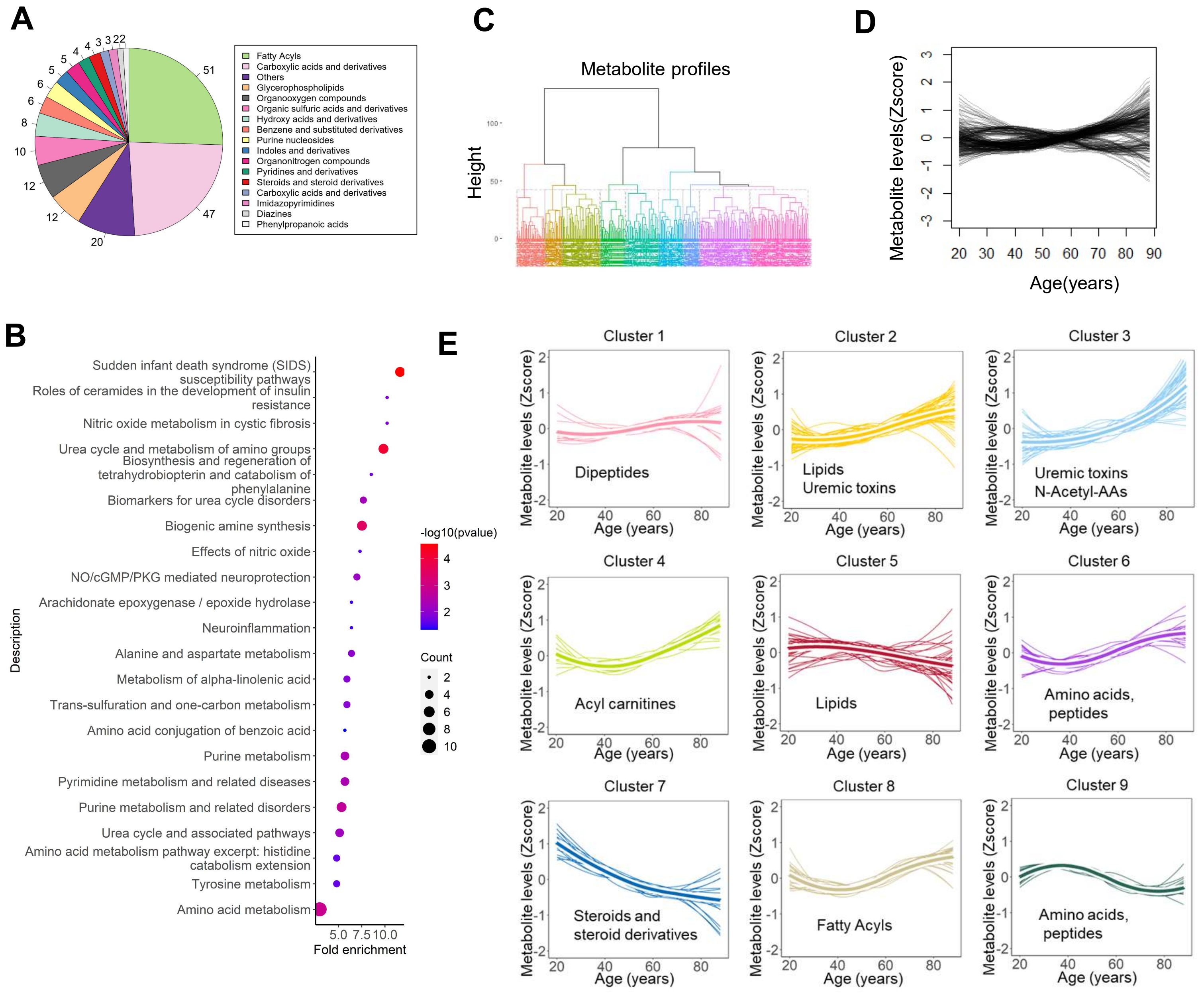

Figure S1

A. Distribution of metabolite classes significantly and positively correlated with aging.

B. Visualization of pathways significantly enriched for metabolites that were significantly and positively correlated with age based on linear modeling. Pathway enrichment was conducted using the wikipathway, enrichment was tested using the hypergeometric test. $P < 0.05$ was shown. Size of circles represents the magnitude of fold enrichment.

C. Unsupervised hierarchical clustering of 280 metabolites significantly correlated with aging based on linear modeling was utilized to group plasma metabolites with similar trajectories, with 9 clusters being identified.

D. Metabolite trajectories across aging. Plasma metabolite levels were z scored, and trajectories of the aging related metabolites were estimated by locally estimated scatterplot smoothing (LOESS) regression.

E. Metabolite trajectories of nine individual clusters identified from unsupervised hierarchical clustering. Clusters were grouped by the similarity of global trajectories. The predominant metabolite classes were presented for each cluster.

Figure S2

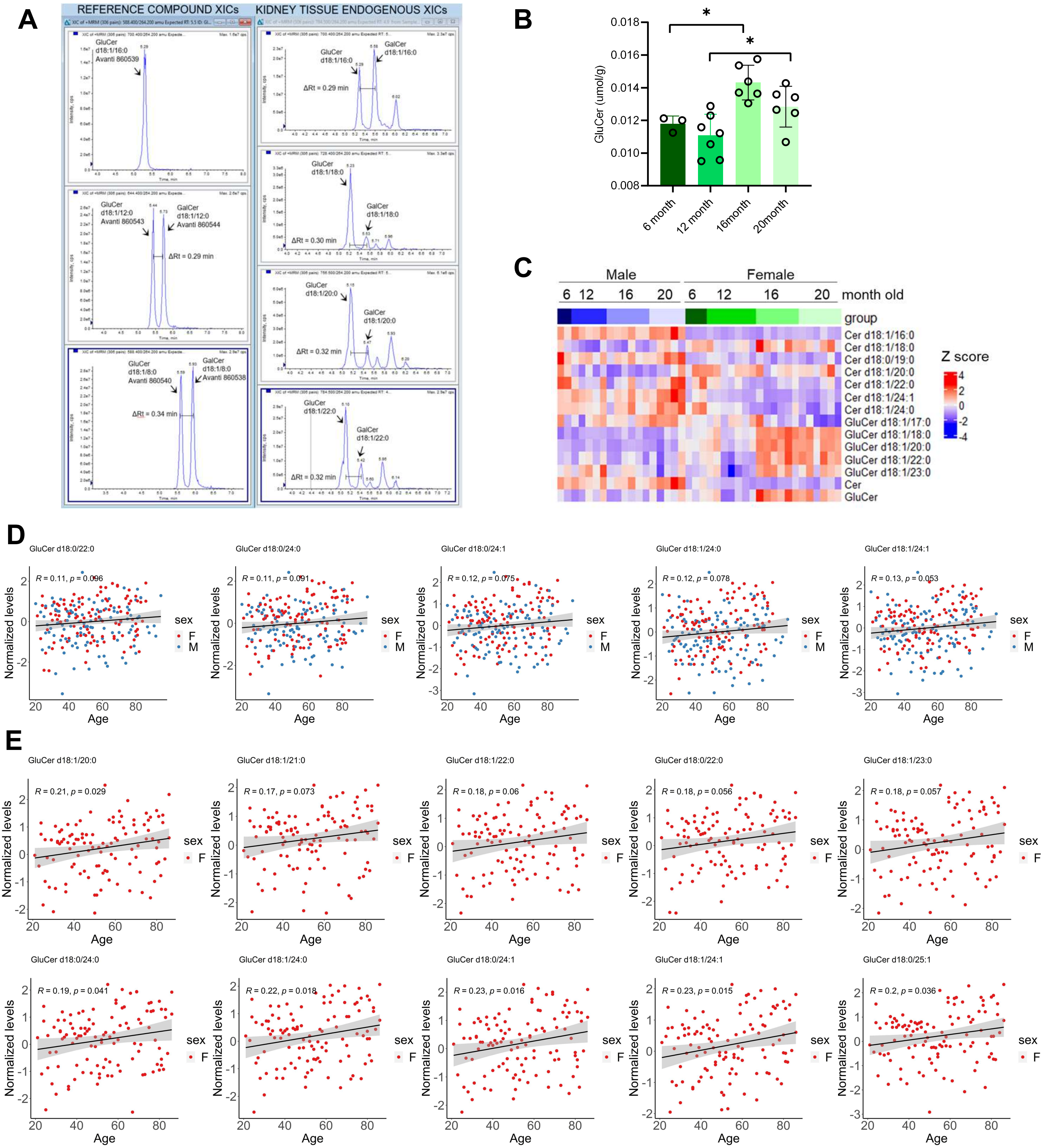

Figure S2

A. XICs displaying clear separation of GluCer and GalCer isomers under our LC gradient. Left panel: Elution of authentic reference compounds. Each pair of GluCer-GalCer isomers are completely separated, with each GluCer species eluting before its GalCer isomer with a difference in central retention time (ΔRt) ≈ 0.30 min. Right panel: Elution of endogenous GluCer and GalCer species in murine kidney tissue lipid extract. C18-C22 GluCers are present in higher endogenous abundances than their corresponding GalCer isomers in female kidney tissues.

B. Total GluCer of female mouse kidneys across normative aging (6 months; n=3, 12 months; n=6, 16 months; n=6 and 20 months; n=6).

C. Heatmap illustrates changes in Cer and GluCer species in male and female kidneys during aging. Kidneys collected from mice across four ages (6 months, 12 months, 16 months and 20 months) were analyzed. Kidney lipid levels were z-scored, and unsupervised hierarchical clustering aggregates lipid species exhibiting similar temporal patterns of changes. Red denote increases while blue denote decreases in lipid levels amongst the groups compared.

D. Correlation of GluCer species in human plasma with age. Measured levels in individual participants were displayed as dots (n = 222), and linear regression coefficients were denoted by black trend lines with confidence intervals shaded in gray, Pearson correlation coefficients between metabolites and age were shown at the top of individual plots. Red dot stands for female and blue dot stands for male.

E. GluCer species from female plasma were significantly correlated with age. Measured levels in individual female participants were displayed as dots (n =111), and linear regression coefficients were denoted by black trend lines with gray confidence intervals. Pearson correlation coefficients between metabolites and age and corresponding P-values were shown at the top of individual plots.

Data are presented as means ± SD; one-way ANOVA followed by Dunnett's post hoc test versus CBE group. *$p < 0.05$. Cer: ceramide; GluCer: glucosylceramide

Figure S3

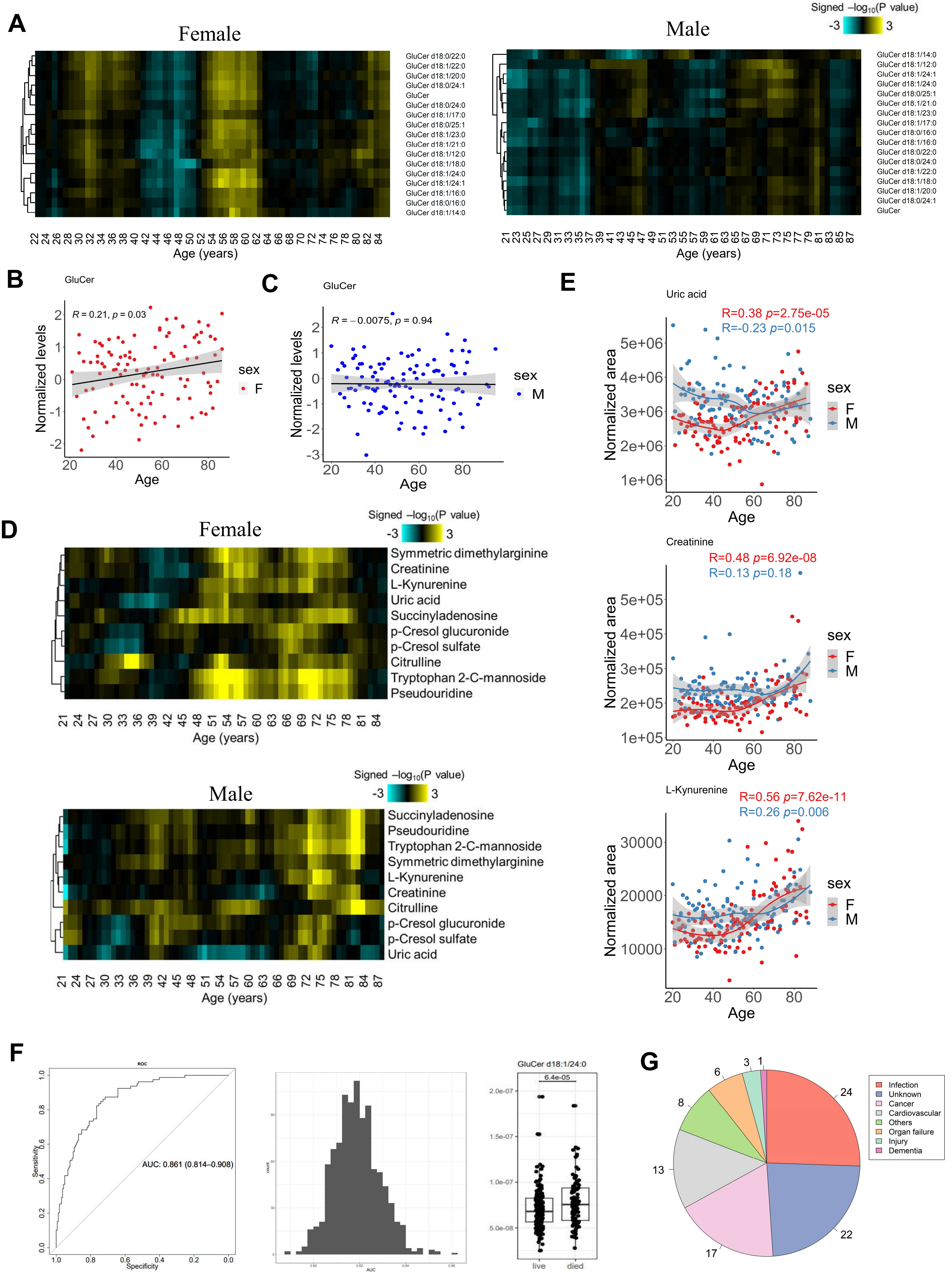
A
Female
Male
Signed −log10(P value)
-3
3
GluCer d18:0/22:0
GluCer d18:1/22:0
GluCer d18:1/20:0
GluCer d18:0/24:1
GluCer
GluCer d18:0/24:0
GluCer d18:1/17:0
GluCer d18:0/25:1
GluCer d18:1/23:0
GluCer d18:1/21:0
GluCer d18:1/12:0
GluCer d18:1/18:0
GluCer d18:1/24:0
GluCer d18:1/24:1
GluCer d18:1/16:0
GluCer d18:0/16:0
GluCer d18:1/14:0
GluCer d18:1/14:0
GluCer d18:1/12:0
GluCer d18:1/24:1
GluCer d18:1/24:0
GluCer d18:0/25:1
GluCer d18:1/21:0
GluCer d18:1/23:0
GluCer d18:1/17:0
GluCer d18:0/16:0
GluCer d18:1/16:0
GluCer d18:0/22:0
GluCer d18:0/24:0
GluCer d18:1/22:0
GluCer d18:1/18:0
GluCer d18:1/20:0
GluCer d18:0/24:1
GluCer
Age (years)
B
GluCer
R = 0.21, p = 0.03
Normalized levels
Age
sex
F
C
GluCer
R = −0.0075, p = 0.94
Normalized levels
Age
sex
M
D
Female
Signed −log10(P value)
Symmetric dimethylarginine
Creatinine
L-Kynurenine
Uric acid
Succinyladenosine
p-Cresol glucuronide
p-Cresol sulfate
Citrulline
Tryptophan 2-C-mannoside
Pseudouridine
Age (years)
Male
Succinyladenosine
Pseudouridine
Tryptophan 2-C-mannoside
Symmetric dimethylarginine
L-Kynurenine
Creatinine
Citrulline
p-Cresol glucuronide
p-Cresol sulfate
Uric acid
Age (years)
E
Uric acid
R=0.38 p=2.75e-05
R=-0.23 p=0.015
Normalized area
Age
sex
F
M
Creatinine
R=0.48 p=6.92e-08
R=0.13 p=0.18
L-Kynurenine
R=0.56 p=7.62e-11
R=0.26 p=0.006
F
ROC
AUC: 0.861 (0.814–0.908)
Sensitivity
Specificity
count
AUC
GluCer d18:1/24:0
6.4e-05
live
died
G
Infection
Unknown
Cancer
Cardiovascular
Others
Organ failure
Injury
Dementia
24
22
17
13
8
6
3
1

Figure S3

A. Plasma GluCer waves across normative aging of human female and male participants characterized by DE-SWAN (n=222). Increases across the sliding age windows were indicated in yellow and decreases in turquoise.

B. Scatter plot of correlation between plasma Glucer in female participants across aging (n=111). Linear regression coefficients were denoted by black trend line with confidence intervals shaded on gray, Pearson correlation coefficients between metabolites and age and the corresponding P-value were shown at the top of the plot.

C. Scatter plot of correlation between plasma Glucer in male participants across aging (n=111). Linear regression coefficients were denoted by black trend line with confidence intervals shaded on gray, Pearson correlation coefficients between metabolites and age and the corresponding P-value were shown at the top of the plot.

D. Temporal waves of ten selected CKD-relevant metabolites across normative aging as characterized by DE-SWAN (n=225) separately for female (upper panel) and male (lower panel) participants. Increases across the sliding age windows were indicated in yellow and decreases in turquoise.

E. Scatter plots illustrating the changes in plasma uric acid, creatine and L-kynurenine across aging. Data from female (red) and male (blue) participants were plotted together. Metabolite trajectories across aging was estimated by LOESS.

F. Lasso models were constructed based on lipid and clinical variables from a longitudinal cohort of elderly people (n=271). ROC curve of the selection with highest accuracy was illustrated and a histogram on the area under curves (AUC) of all individual runs were provided. Barplot shows a comparison between the baseline levels of GluCer d18:1/24:0 in participants who lived or died six years later.

G. Pie-chart illustrates a distribution of the different causes of deaths recorded for the longitudinal cohort of elderly people.

Figure S4

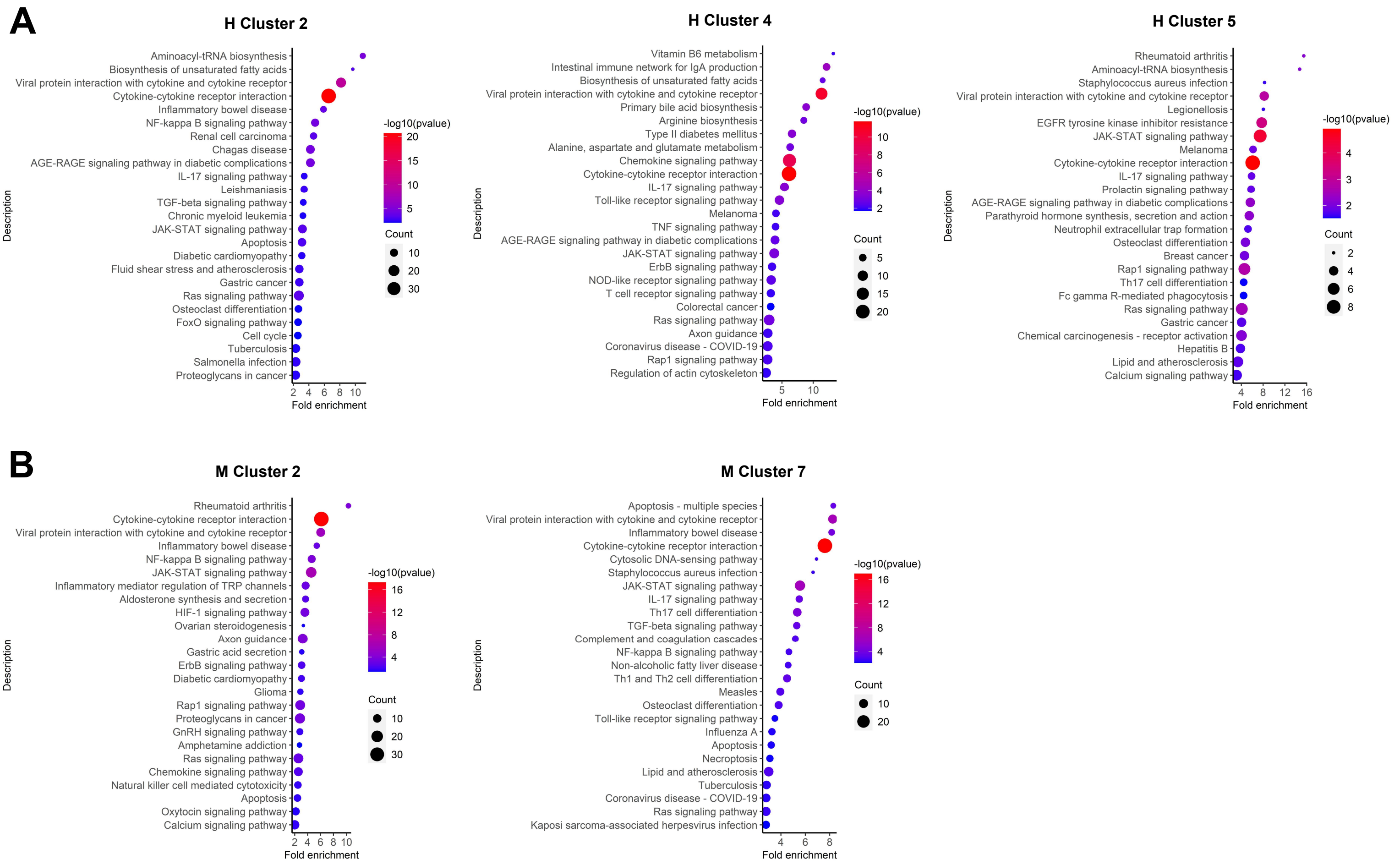

Figure S4

A. KEGG pathway enrichment of the three lipid-protein clusters having similar trajectories with human plasma GluCer across aging (H cluster 2, H cluster 4 and H cluster 5). Top 25 significant pathways of each cluster were shown. Size of circles represents the number of counts. Enrichment was tested using the hypergeometric test and magnitudes of P-values were indicated by the color scale bar.

B. KEGG pathway enrichment of the two lipid-protein clusters having similar trajectories with mouse plasma GluCer across during aging (M cluster 2 and M cluster 7). Top 25 significant pathways of each cluster were shown. Size of circles represents the number of counts. Enrichment was tested using the hypergeometric test and magnitudes of P-values were indicated by the color scale bar.

Figure S5

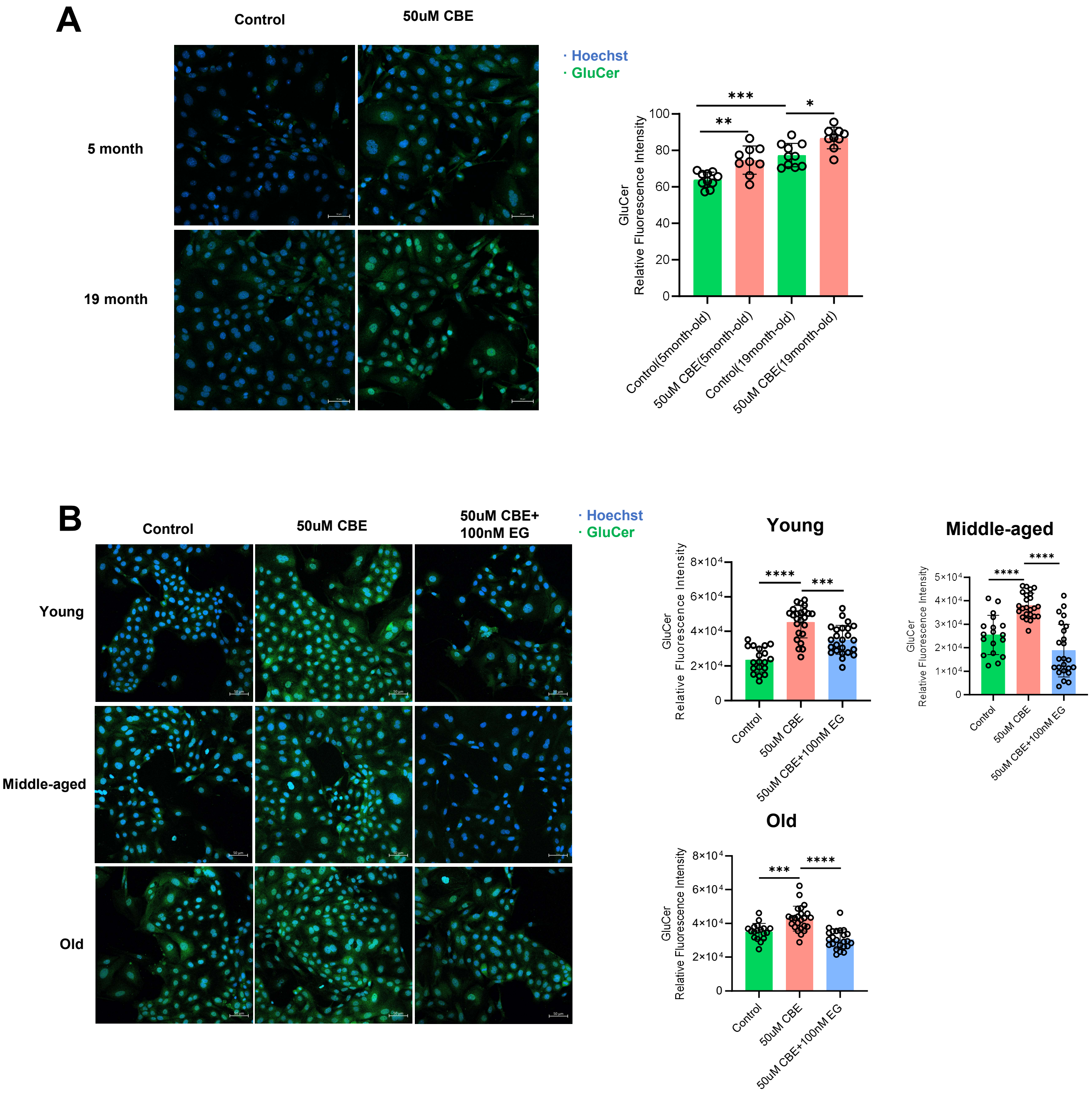

Figure S5. **Representative confocal images of immunocytochemistry of GluCer.**

A. Representative images showing GluCer (green) staining in PCTCs isolated from 5-month-old and 19-month-old female mice were treated with 50 µM CBE for 48h. Quantification of GluCer. Data are presented as means ± SD; One-way ANOVA and Tukey's post hoc test for multiple comparisons. Scale bar=50uM.

B. Representative images showing GluCer (green) staining in PCTCs isolated from young (5-6 months), middle-aged (14-16 months), and old (23-24 months) female mice were treated with 50 µM CBE with or without EG-mediated glucosylceramide synthesis inhibition for 48h. Quantification of GluCer expression from Fig A. Data are presented as means ± SD; One-way ANOVA followed by Dunnett's post hoc test versus CBE group. *p < 0.05, **p < 0.01, ***p < 0.001, ****p < 0.0001. Scale bar=50uM.

Figure S6

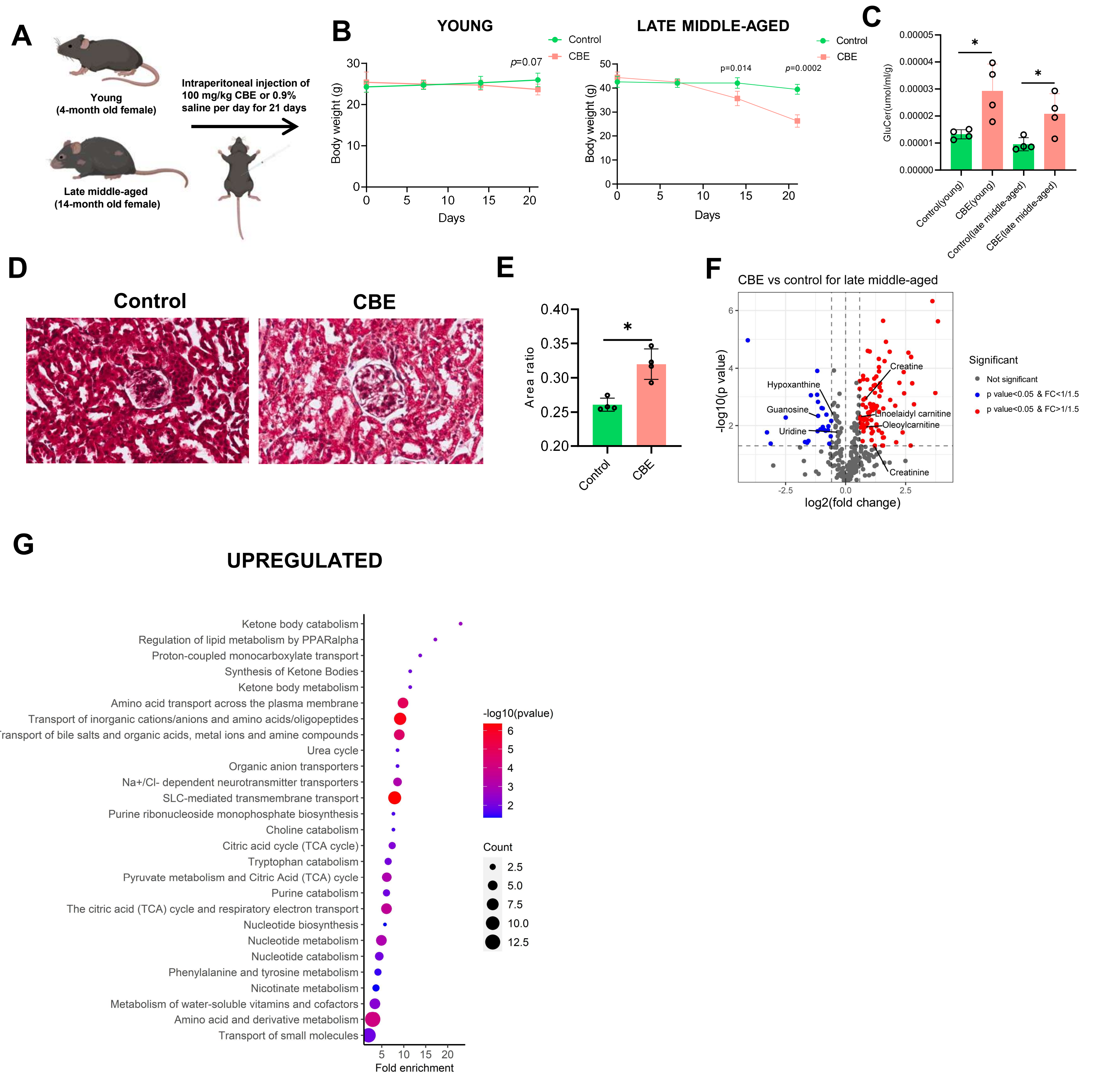

Figure S6. Resiliency metabolic mechanisms maintaining mitochondrial function against GluCer accumulation in late middle-aged female mice.

A. Young (4-month-old) and late middle-aged (14-month-old) female mice were injected intraperitoneally with 100 mg/kg CBE or an equivalent volume of 0.9% NaCl every day for a period of 21 days. At the end of the treatment period, kidneys were harvested for phenotype analysis.

B. Body weights of young (n=4) and late middle-age (n=4) mouse treated with CBE or control saline during the 21-day treatment period. P-values presented were determined using a two-tailed unpaired t-test.

C. GluCer content in the kidneys of 4-month-old and 14-month-old female mice after 21 days of treatment (control 4-month: n=4; CBE-treated 4-month: n=4; control 14-month: n=4 and CBE-treated 14-month: n=4). P-values presented were determined using a two-tailed unpaired t-test.

D. HE staining of mouse kidneys isolated from 14-month late middle-aged female mice injected with saline (n=4) and CBE(n=4).

E. Dotplot of unstained area ratios quantitated from HE staining images of murine kidneys from late middle-aged female mice injected with saline (n=4) and CBE(n=4). P-values presented were determined using a two-tailed unpaired t-test.

F. Volcano plot of metabolite changes in the kidneys of late middle-aged female mice treated with CBE (n=4) compared to control saline-treated group (n=4). Metabolites significantly elevated with CBE treatment (P<0.05, fold-change>|1.5|) were colored in red while metabolites significantly reduced compared to control group in blue. P-values presented were determined using a two-tailed unpaired t-test.

G. Pathways significantly enriched for metabolites elevated in the kidneys of CBE-treated late middle-aged female mice compared to saline-injected control mice based on the Reactome database. Size of circles represents the magnitude of fold enrichment, and magnitude of P-values were indicated by color scale bar. Enrichment was tested using the hypergeometric test.

CBE: conduritol-β-epoxide; HE: Hematoxylin-eosin

Figure S7

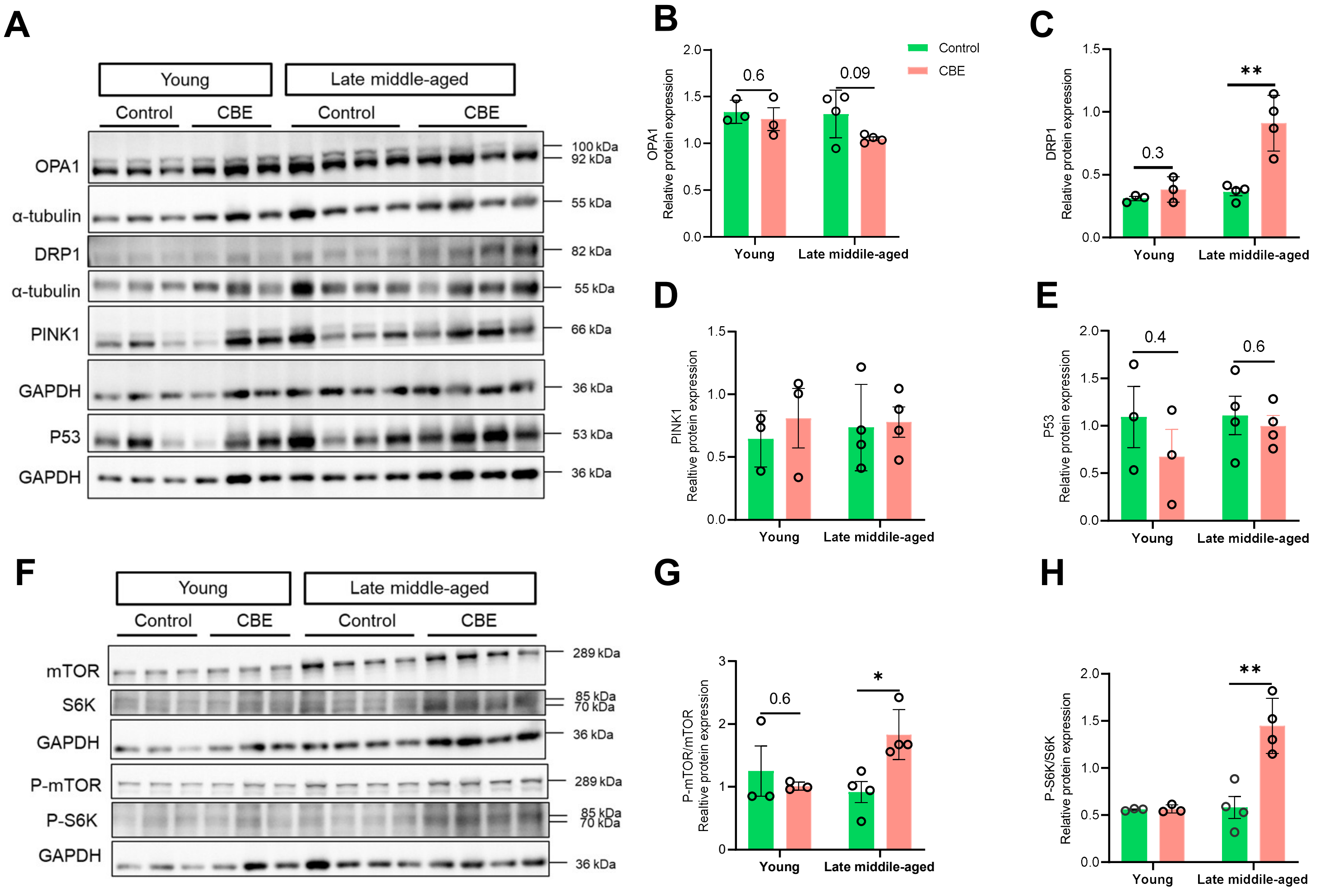

Figure S7. Molecular details of how CBE-induced GluCer accumulation disrupted renal mitochondria dynamics in late middle-aged female mice.

A. Immunoblot analysis on proteins relevant to mitochondrial function and senescence in the kidneys of 4-month-old (young) and 14-month-old (late middle-aged) female mice following 21 days of CBE treatment (control 4-month: n=3; CBE-treated 4-month: n=3; control 14-month: n=4 and CBE-treated 14-month: n=4).

B-E. Barplots on the relative protein expressions of Drp1, Opa1, Pink1 and P53 in the kidneys of 4-month-old (young) and 14-month-old (late middle-aged) female mice after 21 days of treatment.

F. Immunoblot analysis on key proteins involved in the mTOR pathway in the kidneys of 4-month-old (young) and 14-month-old (late middle-aged) female mice following 21 days of CBE treatment (control 4-month: n=3; CBE-treated 4-month: n=3; control 14-month: n=4 and CBE-treated 14-month: n=4).

G-H. Barplots on the ratios of relative protein expressions of p-mTOR/mTOR and p-S6K/S6K in the kidneys of 4-month-old and 14-month-old female mice after 21 days of treatment. Data are presented as means ± SD. Statistical significance was determined using a two-tailed unpaired t-test. *$p < 0.05$, **$p < 0.01$. P53: tumor protein P53; Opa1: Opa1 mitochondrial dynamin like GTPase; GAPDH: glyceraldehyde-3-phosphate dehydrogenase; Drp1: dynamin-related protein 1; Pink1: PTEN-induced kinase 1; mTORC1: mammalian target of rapamycin complex 1; p-mTOR: Ser2448 phosphorylation of mTORC1; S6K: ribosomal S6 kinase; p-S6K: Thr389 phosphorylation of S6K

Figure S8

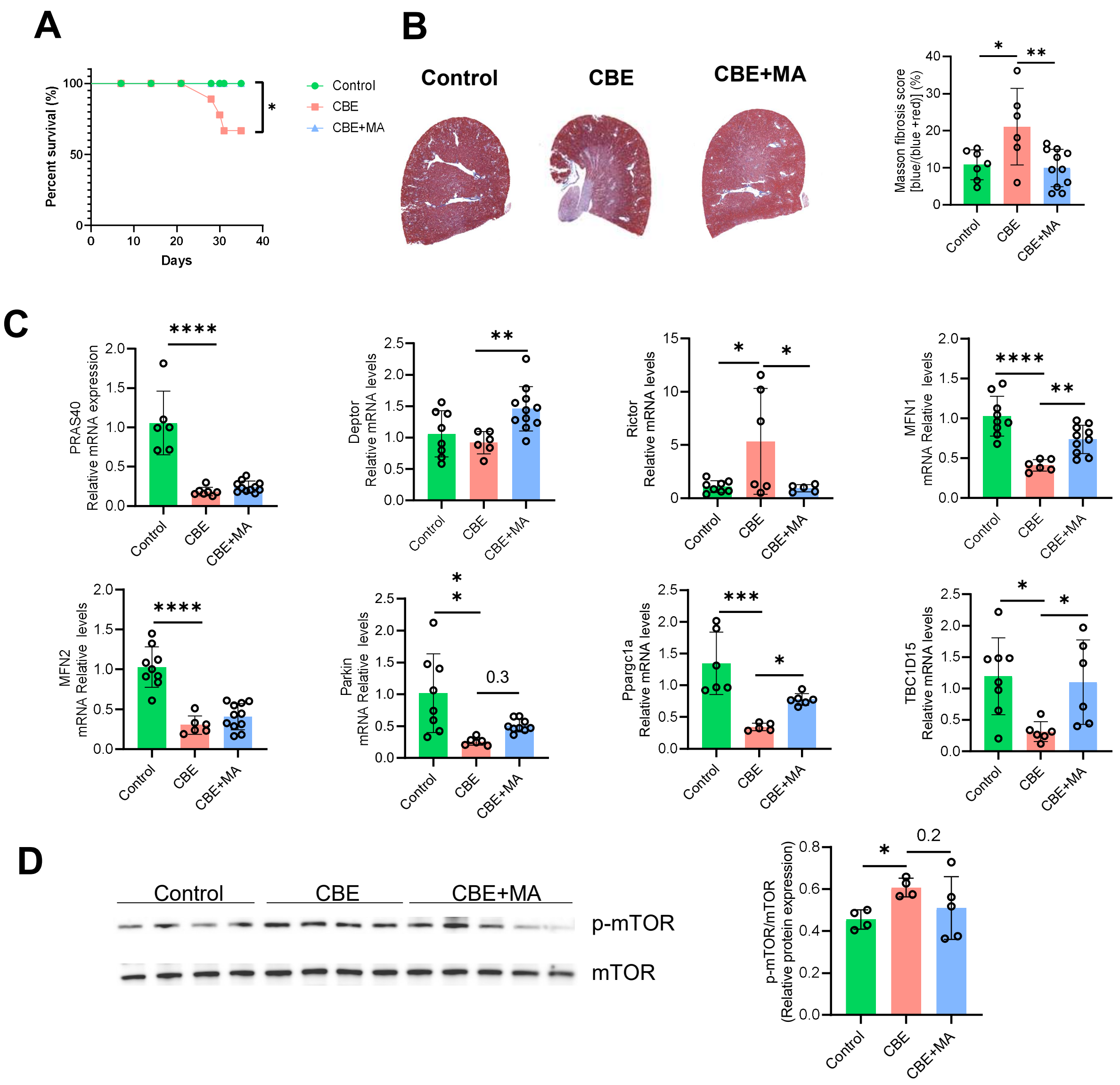

Figure S8. CBE-induced dysregulation of mTOR signaling and mitochondrial dysfunction mitigated by depletion of endogenous purines *in vivo*

Three-month-old male mice were administered 100 mg/kg CBE for 28 days, with one group concurrently receiving 100 mg/kg mycophenolic acid sodium (MA) via gavage (CBE + MA).

(A) Survival curve.

(B) Masson's trichrome staining of kidneys (n=6-11/group). Renal fibrosis area calculated by percentage of blue/(blue + red).

(C) Relative mRNA expression of mTOR pathway regulators and mitochondrial function markers in kidney tissue by qPCR (n=6-11/group).

(D) Western blot for p-mTOR and total mTOR. One-way ANOVA followed by Dunnett's post hoc test versus CBE group. Data are presented as means ± SD. *$p < 0.05$, **$p < 0.01$, ***$p < 0.001$, ****$p < 0.0001$.

Methods

Lipid and metabolite extraction from mouse tissues

Lipids and metabolites were extracted from tissues using established protocols[1, 2]. Briefly, 900 µL of chloroform:methanol (1:2, v/v) containing 10% deionized H_2O was added to each sample. Samples were homogenized on an automated bead ruptor (OMNI, Seattle, WA, USA). Following homogenization, samples were incubated at 1500 rpm at 4 °C for 1 h. At the end of the incubation, 400 µL of ice-cold deionized H_2O and 300 µL of ice-cold chloroform were added to induce phase separation. The samples were centrifuged at 12000 rpm for 5 min at 4 °C. The lower organic phase was transferred to a new tube. A second round of extraction was performed via adding 500 µL of ice-cold chloroform to the remaining aqueous phase. Samples were vortexed and centrifuged at 12000 rpm for 5 min at 4 °C. The lower organic phases from both rounds of extraction were pooled and dried using SpeedVac under OH mode, and reconstituted in chloroform:methanol (1:1; v/v) for lipidomic analyses. Two rounds of extraction were performed to maximise lipid recovery. The remaining aqueous phase containing polar metabolites were centrifuged at 12000 rpm for 5 min at 4 °C. Clean supernatant was transferred into a fresh 1.5 mL centrifuge tube and dried using a SpeedVac (Genevac miVac, Tegent Scientifc Ltd., England). The dried extracts were reconstituted using 2% acetonitrile in water for metabolomics analyses.

Metabolomics

A Waters ACQUITY UPLC HSS T3 column (1.8 µm, 3.0 x 100 mm) (Waters, Dublin, Ireland) was used for separation of polar metabolites. Ultra-performance liquid chromatography (UPLC) (Agilent 1290 II) coupled to the Agilent 6546 LC/Q-TOF (Agilent Technologies, Germany) was used to acquire mass spectrometry (MS) data. The column oven temperature was maintained at 40 °C, and autosampler was set at 10 °C. The injector volume was 5 µL. Flow rate was 0.35 mL min^{-1}. Mobile phase A comprised water containing 0.1% FA (v/v), and mobile phase B was acetonitrile. The following linear gradient was used: 0–1.0 min with 2% B, 1.0–6.0 min with 2%–42% B, 6.0–8.0 min with 42%–65% B, 8.0–10.0 min with 65%–76% B, 10.0–11.0 min with 76%–98% B, 11.0–15.0 min with 98%–98% B. Positive and negative polarity data was acquired on the Agilent 6546 LC/Q-TOF using full scan mode with ranges of m/z 60-1100. Data dependent acquisition using iterative exclusion was conducted via five repeated injections of pooled (QC) plasma sample for each polarity[3]. The MS parameters used for detection were: ESI source voltage +3500 or −3000 kV in positive and negative ion modes, respectively; drying gas temperature, 300 °C; nebulizer pressure, 38psi; nitrogen drying gas, 7L/min; sheath gas temperature and flow at 350°C and 11L/ min, respectively. MS acquisition rate was 2 spectra/sec and m/z data range from 60-1100 were stored in profile mode. Dynamic mass axis calibration was achieved by continuous infusion of a reference mass solution. A cocktail of isotopically-labeled internal standards purchased from the Cambridge Isotope Laboratories were added for metabolite quantitation, including L-Tryptophan-d8, L-Isoleucine-d10, L-leucine-d10, L-Methionine-d3, L-Valine-d8, L-Proline-d7, L-Alanine-d4, DL-Serine-d3, L-Glutamine-d5, L-Aspartic acid-d3, L-Arginine-d7, L-Glutamate-d5, L-Lysine-d9, L-Histidine-13C6, Taurine-13C2, Betaine-d11, Urea-(13C,15N2), L-lactate-d3, Trimethylamine N-oxide-d9, Choline-d13, Malic acid-d3, Citric acid-d4, Succinic acid-d4, Fumaric acid-d2, Hypoxanthine-d3, Xanthine-15N2, Thymidine (13C10,15N2), Inosine-15N4, Cytidine-13C5, Uridine-d2, Methylsuccinic acid-d6, Benzoic acid-d5, Creatine-d3, Creatinine-d3, Glutaric acid-d4, Hippuric acid-d5, Kynurenic acid-d5, L-Citrulline-d4, L-Threonine-(13C4, 15N), L-Tyrosine-d7, P-cresol sulfate-d7, Sarcosine-d3, Trans-4-hydroxy-L-proline-d3, Uric acid-(13C; 15N3), Pyruvate-d3. Peak areas of endogenous metabolites were normalized to the areas of their corresponding isotopically-labeled structural analogues for quantitation[4, 5]. For endogenous metabolites without labeled structural analogues, an automated algorithm selects the optimal internal standard for quantitation based on the rule of minimal coefficients of variations (COVs) after normalization. All detected ions were extracted using the Agilent MassHunter software into Excel in the format of a two-dimensional matrix, including mass

to charge ratio (*m/z*), retention time, and peak areas. MS/MS data were compared with in-house MS/MS spectra obtained from authentic reference compounds, or spectra deposited in the Human metabolome database (HMDB) and METLIN for metabolite identification. A Stein Scott similarity score of > 0.75 was used as cutoff for correct spectral match. Metabolites were annotated and reported using three confidence levels following commonly used community recommendations[6, 7]. Level 1 indicates metabolites confirmed by matching MS1 accurate mass, retention time, and MS/MS spectra to authentic standards analyzed under the same conditions. Level 2 indicates metabolites putatively annotated by matching MS1 and MS/MS spectra to public metabolite spectral libraries without RT confirmation by in-house standards. Level 3 indicates metabolites annotated based on MS1 and MS/MS spectral similarity to known compounds within a chemical class, or based on MS1 features combined with database searches when MS/MS evidence was insufficient. A total of 229 metabolites were annotated from mouse kidney tissues, with 127 metabolites at confidence level 1 (56 %), 92 metabolites at confidence level 2 (40 %) and 10 metabolites at confidence level 3 (4 %) (Table S20).

Lipidomics

Analyses of polar lipids from murine tissue and organ lipid extracts were performed a Jasper HPLC coupled with Sciex Triple Quad 4500MD, whereas neutral lipids were analyzed on an Agilent 1260 HPLC connected to Sciex 5500 QTRAP, both under electrospray ionization mode. Methodological details were comprehensively reported in a recent publication[8]. Internal standard cocktail used for lipid quantification included d9-PC32:0(16:0/16:0), d9-PC36:1p(18:0p/18:1), d7-PE33:1(15:0/18:1), d9-PE36:1p(18:0p/18:1), d31-PS(16:0/18:1), d7-PG33:1(15:0/18:1), d7-PI33:1(15:0/18:1), d7-PA33:1(15:0/18:1), C14-BMP, d9-SM d18:1/18:1, Cer d18:1/d7-15:0, GluCer d18:1/8:0, d3-LacCer d18:1/16:0, Gb3-d18:1/17:0, SL-d18:1/17:0, d7-LPC 18:1, d7-LPE 18:1, LPA-C17:0, LPI-C17:1, LPS-C17:1, LPG-C17:1, DAG(16:0/16:0)-d5, DAG(18:1/18:1)-d5, S1P-d17:1 and Sph-d17:1 obtained from Avanti Polar Lipids, GM3 d18:1/18:0-d3 from Matreya LLC, TAG(14:0)3-d5, TAG(16:0)3-d5, TAG(18:0)3-d5, d6-CE18:0 and d6-cholesterol purchased from CDN Isotopes, and d3-16:0-carnitine from Cambridge Isotope Laboratories. d31-FFA-16:0 from Sigma-Aldrich and d8-FFA-20:4 from Cayman Chemicals were used for quantification of saturated/monounsaturated fatty acids and polyunsaturated fatty acids, respectively.

Polar lipids were separated on a TUP-HB Silica column (3 µm, i.d. 150x2.1 mm) under the following chromatographic conditions: mobile phase A (chloroform:methanol:ammonium hydroxide, 89.5:10:0.5) and mobile phase B (chloroform: methanol: water: ammonium hydroxide:, 27:65:7:1) at a flow rate of 300 µL/min and column oven temperature was at 30℃.The gradient started with 2% B and was held for 2 min, which was then increased to 85% of B over 6 min, and was held at 85% for 1 min before further increasing to 100% B over 0.2 min. The gradient was maintained at 100% B for 3.8 min before returning to 2% B over 0.5 min, and was finally equilibrated at 2% B for 3.5 min prior to the next injection. Glycerol lipids including DAGs and TAGs were quantified using a modified version of reverse phase HPLC/MRM. Separation of neutral lipids were achieved on a Phenomenex Kinetex-C18 column (2.6 µm, i.d. 4.6x100 mm) using an isocratic mobile phase containing chloroform:methanol:0.1 M ammonium acetate 100:100:4 (v/v/v) at a flow rate of 300 µL for 10 min. Levels of short-, medium-, and long-chain TAGs were calculated by referencing to spiked internal standards of TAG(14:0)3-d5, TAG(16:0)3-d5 and TAG(18:0)3-d5 obtained from CDN isotopes (Quebec, Canada), respectively. DAGs were quantified using d5-DAG16:0/16:0 and d5-DAG18:1/18:1 as internal standards from Avanti Polar Lipids. Free cholesterols and cholesteryl esters were quantitated in the atmospheric pressure chemical ionization mode using d6-Cho and d6-CE18:0 (CDN isotopes) as internal standards.

Analysis of GluCer in human plasma extracts were conducted on a Shimadzu Nexera Prominence LC coupled with Sciex QTRAP 7500. GluCer d18:1/8:0 from Avanti Polar Lipids was used as an internal standard for quantification. Separation of GluCer species was performed on a TUP-HB silica column (3

μm, i.d. 150x2.1 mm) under the following chromatographic conditions: mobile phase A (chloroform:methanol:ammonium hydroxide, 89.5:10:0.5) and mobile phase B (chloroform:methanol: ammonium hydroxide: water, 27:65:1:7) at a flow rate of 300 μL/min and column oven temperature at 35°C. The gradient began with 5% of B and was held for 4 min, which was then increased to 35% of B over 1.5 min, and further increasing to 95% B over 3 min. The gradient was maintained at 95% B for 3.5 min before returning to 5% B over 0.5 min, and was finally equilibrated for 4.5 min prior to the next injection.

CBE administration

The GCase inhibitor conduritol-b-epoxide (CBE, MedChem Express) was reconstituted at 10 mg/mL in 0.9% NaCl and stored at -20°C. For cell culture experiments, vehicle or CBE were diluted to the final concentrations as indicated in the figure legends corresponding to individual experiments. For *in vivo* experiments, mice were injected intraperitoneally with 100 mg/kg CBE or an equivalent volume of 0.9% NaCl daily, with injection at alternating sides on each day. Mice were sacrificed at approximately 24 hours following the final injection of CBE or vehicle.

Primary culture of kidney proximal convoluted tubule epithelial cells (PCTCs)

Mice were anesthetized, fixed supine and kidneys were harvested. Kidney cortexes were isolated, minced and digested in 1 mg/mL collagenase at 37°C with shaking for 30 minutes, and filtered successively with 250 μm and 70 μm cell strainers. The filtrates were then centrifuged at 50 x g for 5 min, and the cell pellet was seeded on plates precoated with 20 mM acetic acid and 5 μg collagen type 1 (Thermo Scientific) in mouse renal epithelial cell complete medium (Procell). On the following day, the culture medium was collected and centrifuged at 50 x g for 4 min to pellet cells that had not attached, and the pelleted cells were suspended in fresh growth media and returned to the original culture plate. Proximal tubule epithelial cells were grown to confluence for 4 to 7 days, then used for subsequent experiments as previously described[9].

Measurement of mitochondrial oxygen consumption rate (OCR)

A seahorse XFe96 extracellular flux analyzer (Agilent Technologies) was used to measure oxygen consumption rate (OCR). We evaluated bio-energetic fluxes using a Mito stress Test Kit (Agilent Technologies) according to the manufacturer's protocol. The assay was performed in Agilent Seahorse XF base medium (Agilent Technologies) containing 10 mM glucose, 2 mM glutamine, and 1 mM pyruvate. Mitochondrial function was evaluated by monitoring changes in the OCRs at baseline and after adding 2 μM oligomycin, 1 μM FCCP, and 0.5 μM rotenone/antimycin A. Profiles for mitochondrial function were calculated using the Wave software (Agilent Technologies) as per the manufacturer's instructions.

Immunocytochemistry

Renal tubular epithelial cells were fixed in 4% paraformaldehyde in PBS for 20 min and permeablized with 2% gelatin and 0.1% saponin in PBS at room temperature. Cells were then immuno-labeled with GlcCer (Gly cobiotech, #RAS0011, 1:500). After overnight incubation at 4 °C, coverslips were washed three times with PBS for 5min each, incubated in Alexa-conjugated secondary antibodies for 1 h at room temperature, washed three times. Images were obtained on Leica DMI4000B confocal microscope using Leica Application Suite X.

Kidney histopathology

Kidney samples were fixed in formalin, dehydrated by gradient dehydration, and the sections were paraffin-embedded, then stained with hematoxylin and eosin (H&E) dyes or Matson's trichrome reagent in accordance with the standard procedure, and observed by light microscope[10]. Images were taken with a Zeiss microscope.

HIS-SIM imaging

Renal tubular epithelial cells were seeded at 5,000 cells per well at 37 °C overnight. Cells were treated with 50 μM CBE for 48 h. Following treatment, culture medium was replaced with one of the following fluorescent probes: LysoBrite™ Green (Cayman Chemical, 25154) for lysosome staining, PK MITO Orange (GenVivo, PKMO-1) for mitochondria. Cells were incubated with the respective dyes and imaged using Heissan structured illumination microscopy (HIS-SIM, Guangzhou CSR Biotech) following published protocol[11]. Sparse deconvolution was used to further improve the resolution and contrast in reconstructed images[12].The excitation/emission wavelengths were 450/505 nm for lysosomes, 590/610 nm for mitochondria.

Bioinformatics and statistical analyses

Metabolites peak areas were log-transformed and standardized to z-scores. For metabolite associations with aging, Pearson correlation was calculated with the corr.test function in the R package psych (v.2.1.6). The following linear model: metabolite level ~ $\alpha + \beta 1$ age + ε was used to determine the effect of age on individual metabolite levels. The type II sum of squares was calculated using the ANOVA function of the R package car (v.3.0-11). To estimate metabolite trajectories across aging, plasma metabolite levels were z scored, and locally estimated scatterplot smoothing (LOESS) regression was fitted for each plasma metabolite. To group metabolites with similar trajectories, pairwise differences between LOESS estimates were calculated on the basis of the Euclidian distance, and hierarchical clustering was performed using the complete method. R package clusterProfiler (v.4.10.0) was used for Over-Representation Analysis. KEGG, Wikipathway, Reactome databases are from R package graphite (v.1.44.0). All identified metabolites that can be mapped to specific HMDB IDs were used as the background database for enrichment analysis. DE-SWAN was used to detect non-linear, stage-specific age effects[13]. Instead of fitting a single global age trend, DE-SWAN iteratively centers a sliding age window (e.g., 20 years) at each age and compares molecular levels between two adjacent age parcels within that window (e.g., [age−10, age) vs. [age, age+10]) using a regression model with relevant covariates. By repeating this comparison while sliding the window across the lifespan (typically in 1-year increments), DE-SWAN quantifies local differential changes at each age, enabling the identification of discrete 'waves/crests' where many features shift simultaneously—an approach particularly suitable for capturing non-linear aging trajectories that can be obscured by global linear modeling. Clusters of co-abundant plasma metabolites and lipids were identified using the R package WGCNA as described previously[14, 15]. Time course data was smoothed using loess function from R package. For Trans-omics integration of senescence-associated plasma proteome, metabolome and lipidome was performed using the R package Mfuzz to group proteins and metabolites exhibiting similar temporal patterns across aging into clusters. For the human proteome, lipidome and metabolome data, ages (years) were grouped into seven windows prior to the analysis: 25 (20-29), 35 (30-39), 45 (40-49), 55 (50-59), 65 (60-69), 75 (70-79) and 85 (80-89). Mouse proteome samples were collected at 6, 12, 15 and 20 months, and mouse lipidome samples were analyzed at 6, 12, 16 and 20 months. In order to align the time-points, predicted values based on loess model were obtained at 6, 12, 16 and 20 months prior to data integration. All the statistical analyses were performed using R software version 4.3.2.

References

1. Song, J.W. *et al.* Omics-Driven Systems Interrogation of Metabolic Dysregulation in COVID-19 Pathogenesis. *Cell Metab* **32**, 188-202 e185 (2020).
2. Lam, S.M. *et al.* Quantitative Lipidomics and Spatial MS-Imaging Uncovered Neurological and Systemic Lipid Metabolic Pathways Underlying Troglomorphic Adaptations in Cave-Dwelling Fish. *Molecular Biology and Evolution* **39** (2022).
3. Koelmel, J.P. *et al.* Lipid Annotator: Towards Accurate Annotation in Non-Targeted Liquid Chromatography High-Resolution Tandem Mass Spectrometry (LC-HRMS/MS) Lipidomics Using A Rapid and User-Friendly Software. *Metabolites* **10** (2020).
4. Lam, S.M. *et al.* A multi-omics investigation of the composition and function of extracellular vesicles along the temporal trajectory of COVID-19. *Nat Metab* **3**, 909-922 (2021).
5. Tian, H. *et al.* Precise Metabolomics Reveals a Diversity of Aging-Associated Metabolic Features. *Small Methods* **6**, e2200130 (2022).
6. Schrimpe-Rutledge, A.C., Codreanu, S.G., Sherrod, S.D. & McLean, J.A. Untargeted Metabolomics Strategies—Challenges and Emerging Directions. *J. Am. Soc. Mass Spectrom.* **27**, 1897-1905 (2016).
7. Sumner, L.W. *et al.* Proposed minimum reporting standards for chemical analysis. *Metabolomics* **3**, 211-221 (2007).
8. Miao, H. *et al.* Lipidome Atlas of the Developing Heart Uncovers Dynamic Membrane Lipid Attributes Underlying Cardiac Structural and Metabolic Maturation. *Research* **2022** (2022).
9. Ding, W., Yousefi, K. & Shehadeh, L.A. Isolation, Characterization, And High Throughput Extracellular Flux Analysis of Mouse Primary Renal Tubular Epithelial Cells. *J Vis Exp* (2018).
10. Zhang, X. *et al.* Dietary cholesterol drives fatty liver-associated liver cancer by modulating gut microbiota and metabolites. *Gut* **70**, 761-774 (2021).
11. Huang, X. *et al.* Fast, long-term, super-resolution imaging with Hessian structured illumination microscopy. *Nat. Biotechnol.* **36**, 451-459 (2018).
12. Zhao, W. *et al.* Sparse deconvolution improves the resolution of live-cell super-resolution fluorescence microscopy. *Nat. Biotechnol.* **40**, 606-617 (2021).
13. Lehallier, B. *et al.* Undulating changes in human plasma proteome profiles across the lifespan. *Nat Med* **25**, 1843-1850 (2019).
14. Langfelder, P. & Horvath, S. WGCNA: an R package for weighted correlation network analysis. *BMC Bioinformatics* **9**, 559 (2008).
15. Pedersen, H.K. *et al.* Human gut microbes impact host serum metabolome and insulin sensitivity. *Nature* **535**, 376-381 (2016).
